# Accumulation and Secretion of Coumarinolignans and other Coumarins in *Arabidopsis thaliana* Roots in Response to Iron Deficiency at High pH

**DOI:** 10.3389/fpls.2016.01711

**Published:** 2016-11-23

**Authors:** Patricia Sisó-Terraza, Adrián Luis-Villarroya, Pierre Fourcroy, Jean-François Briat, Anunciación Abadía, Frédéric Gaymard, Javier Abadía, Ana Álvarez-Fernández

**Affiliations:** ^1^Plant Stress Physiology Group, Department of Plant Nutrition, Aula Dei Experimental Station, Consejo Superior de Investigaciones CientíficasZaragoza, Spain; ^2^Biochimie et Physiologie Moléculaire des Plantes, Centre National de la Recherche Scientifique, Institut National de la Recherche Agronomique, Université MontpellierMontpellier, France

**Keywords:** *Arabidopsis*, cleomiscosin, coumarin, fraxetin, iron nutrition, mass spectrometry, root secretion

## Abstract

Root secretion of coumarin-phenolic type compounds has been recently shown to be related to *Arabidopsis thaliana* tolerance to Fe deficiency at high pH. Previous studies revealed the identity of a few simple coumarins occurring in roots and exudates of Fe-deficient *A. thaliana* plants, and left open the possible existence of other unknown phenolics. We used HPLC-UV/VIS/ESI-MS(TOF), HPLC/ESI-MS(ion trap) and HPLC/ESI-MS(Q-TOF) to characterize (identify and quantify) phenolic-type compounds accumulated in roots or secreted into the nutrient solution of *A. thaliana* plants in response to Fe deficiency. Plants grown with or without Fe and using nutrient solutions buffered at pH 5.5 or 7.5 enabled to identify an array of phenolics. These include several coumarinolignans not previously reported in *A. thaliana* (cleomiscosins A, B, C, and D and the 5′-hydroxycleomiscosins A and/or B), as well as some coumarin precursors (ferulic acid and coniferyl and sinapyl aldehydes), and previously reported cathecol (fraxetin) and non-cathecol coumarins (scopoletin, isofraxidin and fraxinol), some of them in hexoside forms not previously characterized. The production and secretion of phenolics were more intense when the plant accessibility to Fe was diminished and the plant Fe status deteriorated, as it occurs when plants are grown in the absence of Fe at pH 7.5. Aglycones and hexosides of the four coumarins were abundant in roots, whereas only the aglycone forms could be quantified in the nutrient solution. A comprehensive quantification of coumarins, first carried out in this study, revealed that the catechol coumarin fraxetin was predominant in exudates (but not in roots) of Fe-deficient *A. thaliana* plants grown at pH 7.5. Also, fraxetin was able to mobilize efficiently Fe from a Fe(III)-oxide at pH 5.5 and pH 7.5. On the other hand, non-catechol coumarins were much less efficient in mobilizing Fe and were present in much lower concentrations, making unlikely that they could play a role in Fe mobilization. The structural features of the array of coumarin type-compounds produced suggest some can mobilize Fe from the soil and others can be more efficient as allelochemicals.

## Introduction

Iron (Fe) is required for many crucial biological processes, and is therefore essential for all living organisms. A sufficient supply of Fe is necessary for optimal plant productivity and agricultural produce quality (Briat et al., 2015). Iron is the fourth most abundant element in the earth’s crust, but its availability for plants is influenced by pH and redox potential, as well as by the concentration of water-soluble Fe-complexes and the solubility of Fe(III)-oxides and oxyhydroxides (Lindsay, 1995). In calcareous soils, which cover more than 30% of the earth surface, the high soil pH and low soil organic matter content lead to Fe concentrations in the bulk soil solution far below those required for the optimal growth of plants and microbes (10^-4^–10^-9^ and 10^-5^–10^-7^ M, respectively; Guerinot and Ying, 1994). Since plants and microbiota have evolved in soils poor in available Fe, they have active mechanisms for Fe acquisition, often relying on the synthesis and secretion of an array of chemicals that modify the neighboring environment and reduce competition for Fe (Crumbliss and Harrington, 2009; Jin et al., 2014; Mimmo et al., 2014; Aznar et al., 2015). Some of these chemicals are capable to mine Fe from the soil *via* solubilization, chelation and reduction processes, whereas others can serve as repellants and/or attractants that inhibit or promote the growth of concomitant organisms.

In plants, two different Fe uptake mechanisms have been characterized (Kobayashi and Nishizawa, 2012). *Graminaceae* species use a chelation-type strategy (Strategy II) based on the synthesis of phytosiderophores (PS), metal-chelating substances of the mugineic acid family: PS are released by roots *via* specific transporters, mine Fe(III) from the soil by forming Fe(III)-PS complexes, and then complexes are taken up by transporters of the Yellow Stripe family. Non-graminaceous species such as *Arabidopsis thaliana* use a reduction-type strategy (Strategy I), based on the reduction of rhizospheric Fe(III) by a Fe(III) chelate reductase (FRO, ferric reduction oxidase) and the uptake of Fe(II) by root plasma membrane transporters (IRT, iron-regulated transporter). Other items of the Strategy I toolbox are an enhanced H^+^-ATPase activity, an increased development of root hairs and transfer cells and the synthesis and secretion into the rhizosphere of a wide array of small molecules, including flavins, phenolic compounds and carboxylates (Cesco et al., 2010; Mimmo et al., 2014). Recent studies have unveiled direct roles in root Fe acquisition for flavin secretion in *Beta vulgaris* (Sisó-Terraza et al., 2016) and phenolics secretion in *Trifolium pratense* (Jin et al., 2006, 2007) and *A. thaliana* (Rodríguez-Celma et al., 2013; Fourcroy et al., 2014, 2016; Schmid et al., 2014; Schmidt et al., 2014).

**FIGURE 1:**
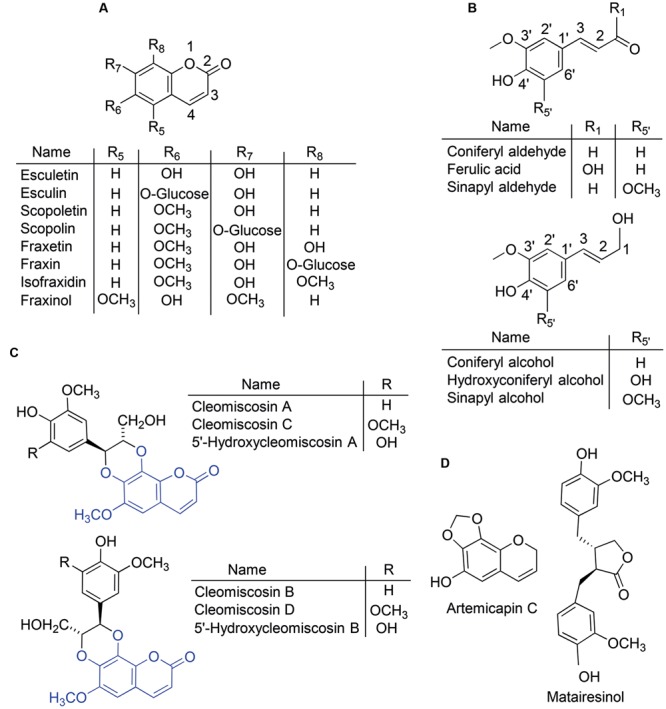
**Chemical structures of some of the phenolic compounds cited in this study.** The plant compounds include coumarins and their glucosides **(A)**, coumarin precursors and monolignols **(B)** and coumarinolignans derived from the coumarin fraxetin **(C)**. The fraxetin moiety is highlighted in blue in the coumarinolignan structures. Compounds used as internal standards **(D)** include a methylenedioxy-coumarin and a lignan.

The phenolic compounds category, including *ca.* 10,000 individual compounds in plants (Croteau et al., 2000), has been long considered to be one of the major components of the cocktail of small molecules secreted by roots of Fe-deficient plants (Cesco et al., 2010). In particular, the coumarin compounds class (O-containing heterocycles with a benzopyrone backbone; **Figure 1A**), which includes at least 1,300 compounds in plants (Borges et al., 2005) has been the focus of recent studies with *A. thaliana*. Upon Fe deficiency, there is a transcriptional up-regulation in roots both of the central phenylpropanoid pathway (from phenylalanine ammonia lyase, one of the upstream enzymes in the pathway, to the coumarate:CoA ligases 4CL1 and 4CL2 that mediate its last step) and of a crucial step of a phenylpropanoid biosynthetic branch, the 2-oxoglutarate-dependent dioxygenase enzyme feruloyl-CoA 6′-hydroxylase1 (F6′H1) (García et al., 2010; Yang et al., 2010; Lan et al., 2011; Rodríguez-Celma et al., 2013; Fourcroy et al., 2014; Schmid et al., 2014; Schmidt et al., 2014), which is responsible for the synthesis of the highly fluorescent coumarin scopoletin (Kai et al., 2008). Up to now, a total of five coumarins, esculetin, fraxetin, scopoletin, isofraxidin and an isofraxidin isomer have been described in Fe-deficient *A. thaliana* roots in both glycoside and aglycone forms (**Figure 1A**, Supplementary Table S1; Fourcroy et al., 2014; Schmid et al., 2014; Schmidt et al., 2014).

Root exudates from Fe-deficient *A. thaliana* plants contain the same coumarins that are found in root extracts, with the aglycone forms being more prevalent (Supplementary Table S1; Fourcroy et al., 2014; Schmid et al., 2014; Schmidt et al., 2014). These exudates have been shown to solubilize 17-fold more Fe from an Fe(III)-oxide (at pH 7.2) when compared to exudates from Fe-sufficient plants, and this was ascribed to the formation of Fe(III)-catechol complexes (Schmid et al., 2014). It is noteworthy that the catechol moiety in two of the five coumarins found to increase with Fe deficiency (esculetin and fraxetin) confers affinity for Fe(III) at high pH and therefore capability for Fe(III) chelation in alkaline soils. In the remaining three coumarins found so far (scopoletin, isofraxidin and its isomer), the catechol moiety is capped *via* hydroxyl (-OH) group methylation (**Figure 1A**), whereas in the glycoside forms of esculetin (esculetin 6-*O*-glucoside, known as esculin) and fraxetin (fraxetin 8-*O*-glucoside, known as fraxin) the catechol is capped *via* hydroxyl group glycosylation (**Figure 1A**). When coumarin synthesis is impaired, as in the *A. thaliana f6′h1* mutant, plants are unable to take up Fe from insoluble Fe sources at high pH (Rodríguez-Celma et al., 2013; Schmid et al., 2014; Schmidt et al., 2014), root exudates are unable to solubilize Fe from insoluble Fe sources, and supplementation of the agarose growth media with scopoletin, esculetin or esculin restores the Fe-sufficient phenotype (Schmid et al., 2014). However, in *in vitro* tests only esculetin (with a catechol moiety), was found to mobilize Fe(III) from an Fe(III) oxide source at high pH (Schmid et al., 2014).

The secretion of coumarins by Fe-deficient roots involves an ABC (ATP-binding cassette) transporter, ABCG37/PDR9, which is strongly over-expressed in plants grown in media deprived of Fe (Yang et al., 2010; Fourcroy et al., 2014, 2016) or containing insoluble Fe(III) at high pH (Rodríguez-Celma et al., 2013). The export of scopoletin, fraxetin, isofraxidin, and an isofraxidin isomer was greatly impaired in the mutant *abcg37* (Fourcroy et al., 2014), which, as it occurs with *f6′h1*, is inefficient in taking up Fe from insoluble Fe(III) at pH 7.0 (Rodríguez-Celma et al., 2013). The root secretion of fluorescent phenolic compounds in *A. thaliana* also requires the Fe deficiency-inducible β-glucosidase BGLU42 (Zamioudis et al., 2014). On the other hand, the IRT1/FRO2 high-affinity root Fe uptake system is necessary for the plant to take up Fe once mobilized, since *irt1* and *fro2* plants grown with unavailable Fe and in presence of phenolics develop chlorosis (Fourcroy et al., 2016). The co-regulation of *ABCG37* and coumarin synthesis genes with *FIT, IRT1, FRO2* and *AHA2* (Rodríguez-Celma et al., 2013) as well as the requirement of *FIT* for *F6’H1* up-regulation upon Fe deficiency (Schmid et al., 2014) support that all these components act in a coordinated mode.

Limitations inherent to the analytical procedures used and/or difficulties in compound structure elucidation have prevented the full characterization of the changes in coumarin composition promoted by Fe deficiency. First, HPLC coupled to fluorescence detection and mass spectrometry (MS and MS^n^) identification was used, therefore focusing only on fluorescent coumarin compounds changing in response to Fe deficiency (Fourcroy et al., 2014); a similar approach was taken later on by Schmid et al. (2014). In a second approach, the use of full chromatographic MS profiles permitted the detection of dozens of compounds changing with Fe deficiency, but only the same coumarins already found with the fluorescence detection approach could be identified (Schmidt et al., 2014).

The aim of this study was to gain insight into the phenolic composition of *A. thaliana* root exudates in response to Fe deficiency, a necessary step for a thorough understanding of the function of phenolics in plant Fe acquisition. Root extracts and exudates from Fe-sufficient and Fe-deficient *A. thaliana* plants grown at pH 5.5 and 7.5 have been analyzed by HPLC coupled to five different detectors: fluorescence, photodiode array, MS-time of flight (TOF), MS-ion trap and MS-MS tandem quadrupole (Q)-TOF, and identification and quantification of phenolics was carried out in roots and exudates. Up to now, quantification of coumarins in roots and exudates from Fe-deficient *A. thaliana* plants had been done only for the two fluorescent compounds esculetin and scopoletin (Schmid et al., 2014). We report herein the identification and quantification of coumarinolignans, coumarin precursors and additional coumarin glycosides, among an array of phenolics accumulated and/or secreted by *A. thaliana* roots in response to Fe deficiency. The root accumulation and secretion of coumarins and coumarinolignans was much higher in plants grown at pH 7.5 than those grown at pH 5.5, and the catechol coumarin fraxetin was predominant in nutrient solutions but not in root extracts. These findings demonstrate the inherent chemical complexity involved in the survival of *A. thaliana* in conditions of high competition for Fe, and give clues for the possible roles of some of the phenolic compounds found.

## Materials and Methods

### Plant Culture and Experimental Design

*Arabidopsis thaliana* (L.) Heynh (ecotype Col0) seeds were germinated, pre-grown and grown as indicated in Fourcroy et al. (2014) with several modifications. Germination and plant growth took place in a controlled environment chamber (Fitoclima 10000 EHHF, Aralab, Albarraque, Portugal), at 21°C, 70% relative humidity and a photosynthetic photon flux density of 220 μmol m^-2^ s^-1^ photosynthetic active radiation with a photoperiod of 8 h light/16 h dark. Seeds were sown in 0.2 ml tubes containing 0.6% agar prepared in nutrient solution 1/4 Hoagland, pH 5.5. Iron was added as 45 μM Fe(III)-EDTA. After 10 d in the growth chamber, the bottom of the tubes containing seedlings was cut off and the tubes were placed in opaque 300-ml plastic boxes (pipette tip racks; Starlab, Hamburg, Germany), containing aerated nutrient solution 1/2 Hoagland, pH 5.5, supplemented with 20 μM Fe(III)-EDTA. Plants were grown for 11 d and nutrient solutions were renewed weekly. After that, plants (12 plants per rack) were grown for 14 days in nutrient solution 1/2 Hoagland with 0 or 20 μM Fe(III)-ethylendiaminedi(*o*-hydroxyphenylacetate) [Fe(III)-EDDHA; Sequestrene, Syngenta, Madrid, Spain]. Solutions were buffered at pH 5.5 (with 5 mM MES) or at 7.5 (with 5 mM HEPES) to maintain a stable pH during the whole treatment period. Nutrient solutions were renewed weekly. Two batches of plants were grown and analyzed. Pots without plants, containing only aerated nutrient solution (with and without Fe) were also placed in the growth chamber and the nutrient solutions sampled as in pots containing plants; these samples were later used as blanks for root exudate analyses.

Roots were sampled 3 days after the onset of Fe deficiency treatment, immediately frozen in liquid N_2_, and stored at -80°C for RNA extraction. Nutrient solutions were sampled at days 7 and 14 after the onset of Fe deficiency treatment, and immediately stored at -20°C until extraction of phenolic compounds. Shoots and roots were sampled separately at the end of the experimental period. Leaf disks (0.1 cm × 0.1 cm) were taken from young leaves and stored at -20°C for photosynthetic pigment analysis. Roots were washed with tap water and then with type I water, dried with filter paper, and then frozen immediately (in aliquots of approximately 300 mg) in liquid N_2_ and stored at -80°C until extraction of phenolic compounds. Roots and shoots from 12 plants per treatment and replication were processed for mineral analysis as in Fourcroy et al. (2014).

### Photosynthetic Pigment Composition

Leaf pigments were extracted with acetone in the presence of Na ascorbate and stored as described previously (Abadía and Abadía, 1993). Pigment extracts were thawed on ice, filtered through a 0.45 μm filter and analyzed by HPLC-UV/visible as indicated in Larbi et al. (2004), using a HPLC apparatus (600 pump, Waters, Mildford, MA, USA) fitted with a photodiode array detector (996 PDA, Waters). Pigments determined were total chlorophyll (*Chl a* and *Chl b*), neoxanthin, violaxanthin, taraxanthin, antheraxanthin, lutein, zeaxanthin and β-carotene. All chemicals used were HPLC quality.

### Mineral Analysis

Plant tissues were ground and digested as indicated in Fourcroy et al. (2014). Iron, Mn, Cu, and Zn were determined by flame atomic absorption spectrometry using a SOLAAR 969 apparatus (Thermo, Cambridge, UK).

### Extraction of Phenolic Compounds from Roots and Nutrient Solutions

Phenolic compounds were extracted from roots and nutrient solutions as described in Fourcroy et al. (2014), with some modifications. First, extraction was carried out without adding internal standards (IS) to identify relevant compounds, including those increasing (or appearing) with Fe deficiency. This extract was also used to check for the presence of the compounds used as IS and other endogenous isobaric compounds that may co-elute with them, since in both cases there will be analytical interferences in the quantification process. The extraction was then carried out adding the following three IS compounds: artemicapin C (**Figure 1D**), a methylenedioxy-coumarin, for quantification of the coumarins scopoletin, fraxetin, isofraxidin and fraxinol; esculin (**Figure 1A**), the glucoside form of the coumarin esculetin, for quantification of coumarin glycosides; and the lignan matairesinol (**Figure 1D**), for quantification of coumarinolignans.

Frozen roots (*ca*. 100 mg) were ground in liquid N_2_ using a Retsch M301 ball mill (Restch, Düsseldorf, Germany) for 3 min and then phenolic compounds were extracted with 1 ml of 100% LC-MS grade methanol, either alone or supplemented with 20 μl of a IS solution (37.5 μM artemicapin C, 50 μM esculin and 37.5 μM matairesinol) by homogenization in the same mill for 5 min. The supernatant was recovered by centrifugation (12,000 × *g* at 4°C and 5 min), and stored at -20°C. The pellet was re-suspended in 1 ml of 100% methanol, homogenized again for 5 min and the supernatant recovered. The two supernatant fractions were pooled, vacuum dried in a SpeedVac (SPD111V, Thermo-Savant, Thermo Fisher Scientific, Waltham, Massachusetts, MA, USA) and dissolved with 250 μl of a solution containing 15% methanol and 0.1% formic acid. Extracts were filtered through poly-vinylidene fluoride (PVDF) 0.45 μm ultrafree-MC centrifugal filter devices (Millipore) and stored at -80°C until analysis.

Phenolic compounds in the nutrient solutions (300 ml of solution used for the growth of 12 plants) were retained in a SepPack C_18_ cartridge (Waters), eluted from the cartridge with 2 ml of 100% LC-MS grade methanol, and the eluates stored at -80°C. Samples were thawed and a 400 μl aliquot was dried under vacuum (SpeedVac) alone or supplemented with 10 μl of a IS solution (80 μM artemicapin C and 150 μM matairesinol). Dried samples were dissolved in 15% methanol and 0.1% formic acid to a final volume of 100 μl, and then analyzed by HPLC-MS. No determinations could be made in nutrient solutions of Fe-sufficient plants due to the presence of Fe(III)-EDDHA, that causes the overloading of C_18_ materials.

### Extraction of Cleomiscosins from *Cleome viscosa* Seeds

Cleomiscosins were extracted from *Cleome viscosa* seeds (B & T World Seeds, Paguignan, France) as described by Chattopadhyay et al. (2008). Seeds were ground using a Retsch M400 ball mill and 25 g of the powder was defatted by homogenization with 50 ml petroleum ether at 25°C for 48 h. The defatting procedure was repeated three times. The solid residue was extracted with 50 ml methanol for 48 h at 25°C, and the extraction was repeated three times. The methanolic extracts were pooled, dried with a rotavapor device and the residue dissolved in 15% methanol and 0.1% formic acid.

### Phenolic Compounds Analysis by HPLC-Fluorescence and HPLC-UV/VIS/ESI-MS(TOF)

HPLC-fluorescence analyses were carried out using a binary HPLC pump (Waters 125) coupled to a scanning fluorescence detector (Waters 474) as in Fourcroy et al. (2014). Separations were performed using an analytical HPLC column (Symmetry^®^ C_18_, 15 cm × 2.1 mm i.d., 5 μm spherical particle size, Waters) protected by a guard column (Symmetry^®^ C_18_, 10 mm × 2.1 mm i.d., 3.5 μm spherical particle size, Waters) and a gradient mobile phase built with 0.1% (v/v) formic acid in water and 0.1% (v/v) formic acid in methanol (Elution program 1; Supplementary Table S2). The flow rate and injection volume were 0.2 ml min^-1^ and 20 μl, respectively. Phenolic compounds were detected using λ_exc_ 365 and λ_em_ 460 nm.

HPLC-UV/VIS/ESI-MS(TOF) analysis was carried out with an Alliance 2795 HPLC system (Waters) coupled to a UV/VIS (Waters PDA 2996) detector and a time-of-flight mass spectrometer [MS(TOF); MicrOTOF, Bruker Daltonics, Bremen, Germany] equipped with an electrospray (ESI) source. Two HPLC protocols were used, the one described above and a second one with a different elution program (Elution program 2; Supplementary Table S2) designed to improve the separation of the phenolic compounds of interest. The ESI-MS(TOF) operating conditions and software used were as described in Fourcroy et al. (2014). Mass spectra were acquired in positive and negative ion mode in the range of 50–1000 mass-to-charge ratio (*m/z*) units. The mass axis was calibrated externally and internally using Li-formate adducts [10 mM LiOH, 0.2% (v/v) formic acid and 50% (v/v) 2-propanol]. The internal mass axis calibration was carried out by introducing the calibration solution with a divert valve at the first and last 3 min of each HPLC run. Molecular formulae were assigned based on exact molecular mass with errors <5 ppm (Bristow, 2006). Phenolic standards used are shown in Supplementary Table S3. Concentrations of phenolic compounds were quantified using external calibration with internal standardization with the exception of ferulic acid hexoside and the cleomiscosins. Ferulic acid hexoside was quantified as fraxin because there is no commercially available authenticated standard. The levels of the cleomiscosins are expressed in peak area ratio, relative to the lignan matairesinol used as IS. For quantification, analytes and IS peak areas were obtained from chromatograms extracted at the *m/z* (±0.05) ratios corresponding to [M+H]^+^ ions, with the exception of glycosides, where the *m/z* ratios corresponding to [M-hexose+H]^+^ ions were used.

### Phenolic Compounds Analysis by HPLC/ESI-MS(Q-TOF) and by HPLC/ESI-MS(Ion Trap)

Phenolic compounds were also analyzed by HPLC/ESI-MS(Q-TOF) using a 1100 HPLC system (Agilent Technologies) coupled to a quadrupole time-of-flight mass spectrometer (Q-TOF; MicroTOF-Q, Bruker Daltonics) equipped with an ESI source. The HPLC conditions were described in Fourcroy et al. (2014) (see above and Supplementary Table S2). The ESI-MS(Q-TOF) operating conditions were optimized by direct injection of 50 μM solutions of phenolic compound standards at a flow rate of 250 μl h^-1^. Mass spectra (50–1000 *m/z* range) were acquired in positive ion mode, with capillary and endplate offset voltages of 4.5 and -0.5 kV, respectively, and a collision cell energy of 100–2000 eV. The nebulizer (N_2_) gas pressure, drying gas (N_2_) flow rate and drying gas temperature were 1.0 bar, 4.0 L min^-1^ and 200°C, respectively. The mass axis was calibrated externally and internally as indicated above for the HPLC/ESI-MS(TOF) analysis. Molecular formulae for the product ions were assigned based on exact molecular mass with errors <5 ppm (Bristow, 2006).

HPLC/ESI-MS(ion trap) analysis was carried out with an Alliance 2795 HPLC system (Waters) coupled to an ion-trap mass spectrometer (HCT Ultra, BrukerDaltonics) equipped with an ESI source. The HPLC conditions were as described in Fourcroy et al. (2014) and Supplementary Table S2 (Elution program 2). ESI-ion trap-MS analysis was carried out in positive and/or negative ion mode, the MS spectra were acquired in the standard mass range mode and the mass axis was externally calibrated with a tuning mix (Agilent). The HCT Ultra was operated with settings shown in Supplementary Table S4. The ions of interest were subjected to collision induced dissociation (CID; using the He background gas present in the trap for 40 ms) to produce a first set of fragment ions, MS/MS or MS^2^. Subsequently, some of the fragment ions were isolated and fragmented to give the next set of fragment ions, MS^3^ and so on. For each precursor ion, fragmentation steps were optimized by visualizing fragment intensity changes.

### RNA Extraction and Quantitative RT-PCR Analysis

Total RNA was extracted from roots using the RNeasy Plant Mini Kit (Quiagen). One microgram RNA was treated with RQ1 DNase (Promega) before use for reverse transcription (Goscript reverse transcriptase; Promega) with oligo (dT)18 and 0.4 mM dNTPs (Promega). The cDNAs were diluted twice with water, and 1 μl of each cDNA sample was assayed by qRT-PCR in a LightCycler 480 (Roche Applied Science) using Lightcycler 480 SYBR Green master I (Roche Applied Science). Expression levels were calculated relative to the housekeeping gene PP2 (At1g13320) using the ΔΔCT method to determine the relative transcript level. The primers used for qRT-PCR were those described in Fourcroy et al. (2014) and indicated in Supplementary Table S5.

### Dissolution of Fe(III)-oxide Using Coumarins

Ten milligrams of poorly crystalline Fe(III)-oxide was incubated (in the dark at 25°C and 300 ppm in a Eppendorf Thermomixer Comfort, Eppendorf AG, Hamburg, Germany) for 6 h with 1.5 ml of an assay solution containing appropriated concentrations (in the range of 0–100 μM) of different coumarins (fraxin, fraxetin, scopoletin, and isofraxidin) and 600 μM of bathophenanthrolinedisulphonate (BPDS) -as Fe(II) trapping agent- and buffered at pH 5.5 (with 5 mM MES-KOH) or pH 7.5 (with 5 mM HEPES-KOH). Afterward, the assay medium was filtered through PVDF 0.22 μm centrifugal filters (Millipore) at 10,000 *g* for 1 min. Absorbance was measured at 535 nm in the filtrates and then the Fe(II) concentration determined as Fe(II)-BPDS_3_ using an extinction coefficient of 22.14 mM^-1^ cm^-1^. The filtrates were also measured for total Fe by Inductively Coupled Plasma Mass Spectrometry (ICP-MS, Agilent 7500ce, Santa Clara, CA, USA) after diluting a 50 μl aliquot with 65% ultrapure HNO_3_ (TraceSELECT Ultra, Sigma–Aldrich).

### Statistical Analyses

Statistical analysis was carried out with SPSS for PC (v.23.0, IBM, Armonk, NY, USA), using ANOVA or non-parametric tests (*p* ≤ 0.05), and a Levene test for checking homogeneity of variances. *Post hoc* multiple comparisons of means corresponding to each one the four different treatments were carried out (*p* ≤ 0.05) using Duncan test when variances were equal and Games–Howell’s test when variances were unequal.

## Results

### Changes in Leaf Photosynthetic Pigment Concentrations, Fe Contents and Biomass with Fe Deficiency and High pH

**FIGURE 2:**
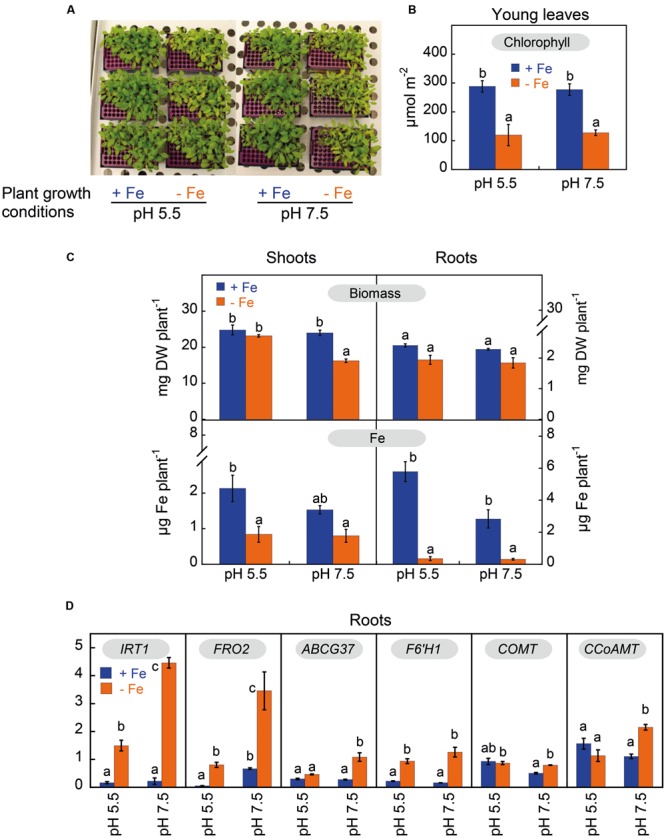
**Effects of Fe deficiency and high pH on plant Fe status, root Fe uptake machinery and phenylpropanoid pathway components in *Arabidopsis thaliana*.** Plants were pre-grown for 11 days in the presence of 20 μM Fe (III)-EDTA at pH 5.5, and then grown for 14 days in a medium with 0 (-Fe) or 20 μM (+Fe) Fe(III)-EDDHA in nutrient solutions buffered at pH 5.5 (with 5 mM MES-NaOH) or 7.5 (with 5mM HEPES-NaOH). **(A)** Plants at day 14 after imposing treatments. **(B)** Leaf chlorophyll concentration in young leaves of plants at day 14 after imposing treatments; data are means ± SE (*n* = 3) and significant differences among treatments (at *p* < 0.05) are marked with different letters above the columns. **(C)** Dry weights and Fe contents in shoots and roots at day 14 after imposing treatments. Data are means ± SE for biomass (*n* = 5) and for Fe contents (*n* = 2–5), and significant differences among treatments (at *p* < 0.05) are marked with different letters above the columns. **(D)** Abundance of *IRT1, FRO2, ABCG37* (*PDR9*), *F6’H1, COMT* and *CCoAMT* transcripts in roots at day 3 after imposing treatments. RNAs were extracted from roots and analyzed by qRT-PCR, using PP2 (At1g13320) as housekeeping gene. The ΔΔCT method was used to determine the relative transcript level. Data are means ± SE (*n* = 3–5). For each gene, significant differences among treatments (at *p* < 0.05) are marked with different letters above the columns.

*Arabidopsis thaliana* plants grown for 14 days in zero-Fe nutrient solution, buffered at either pH 5.5 or pH 7.5, had visible symptoms of leaf chlorosis (**Figure 2A**). The Chlorophyll (*Chl*) concentration in young leaves decreased by 56% in response to Fe deficiency, but was unaffected by the nutrient solution pH (**Figure 2B**). The concentrations of other photosynthetic pigments (neoxanthin, violaxanthin, lutein and β-carotene) in young leaves also decreased upon Fe deficiency (in the range of 48–60%) and were unaffected by the plant growth pH (Supplementary Table S6).

Iron deficiency decreased shoot biomass by 32% only when plants were grown at pH 7.5, whereas root biomass did not change significantly (**Figure 2C**). Shoot Fe content decreased significantly with Fe deficiency only in plants grown at pH 5.5 (by 61%; **Figure 2C**), whereas root Fe content was markedly decreased by 92% in plants grown at both pH values (**Figure 2C**). Iron deficiency also affected the contents of other micronutrients in plants, and this occurred mainly in shoots (Supplementary Table S7). The largest change found was a sixfold increase over the control value in the shoot Cu content of plants grown at pH 5.5.

### Changes in the Expression of Genes Involved in Fe Root Uptake and the Phenylpropanoid Pathway with Fe Deficiency and High pH

The transcript levels of *IRT1, FRO2, ABCG37, F6’H1*, the caffeic acid/5-hydroxyferulic acid *O*-methyltransferase (*COMT*) and the trans-caffeoyl-CoA 3-*O*-methyltransferase (*CCoAMT*) were assessed by quantitative RT-PCR in control (Fe-sufficient) and Fe-deficient roots from both plants grown at pH 5.5 or at pH 7.5 3 days after treatment onset (**Figure 2D**). Under high Fe supply, the only pH effect observed was for *FRO2*, whose transcript abundance was 12-fold higher in plants grown at pH 7.5 than in those grown at pH 5.5. Under Fe deficiency conditions, *IRT1* and *FRO2* gene expression increased in plants grown both at pH 5.5 and pH 7.5; the increases were ninefold for *IRT1* and 15-fold for *FRO2* in plants grown at pH 5.5, and 20-fold for *IRT1* and 5-fold for *FRO2* in plants grown at pH 7.5. Other genes studied, *ABCG37* and *F6’H1*, also showed increases in their expression in response to Fe deficiency when compared to the Fe-sufficient controls, although they were smaller than those observed for *IRT1* and *FRO2*. The increases in *ABCG37* gene expression were 2- (although this change was not statistically significant) and 4-fold in plants grown at pH 5.5 and pH 7.5, respectively, whereas those of *F6’H1* were 4- and 8-fold in plants grown at pH 5.5 and pH 7.5. On the other hand, *COMT* and *CCoAMT* gene expression in roots was only increased by Fe deficiency at pH 7.5 (twofold).

### *Arabidopsis* Roots Accumulate and Secrete an Array of Fluorescent and Non-fluorescent Phenolic-Type Compounds with Fe Deficiency and High pH

Methanolic extracts of roots of *A. thaliana* plants and their nutrient solutions were analyzed using the reverse phase C_18_ HPLC-based method used in Fourcroy et al. (2014) (Elution program 1), using both UV/VIS detection in the range 200–600 nm and fluorescence detection at λ_exc_ 365 and λ_em_ 460 nm (only the latter was used in the original study). Fluorescence alone cannot detect all phenolic compounds, since many of them emit little or no fluorescence. However, all phenolic compounds absorb light in the UV region; coumarins, their derivatives and precursors (e.g., ferulic and other cinnamic acids) have absorption maxima in the range 290–330 nm.

**FIGURE 3:**
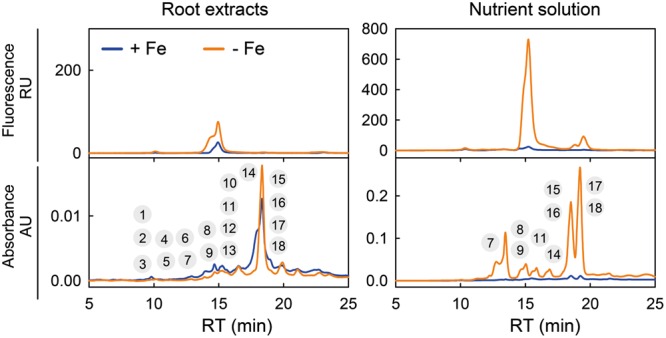
**Chromatographic separation of a range of phenolic-type compounds produced in response to Fe deficiency by *Arabidopsis thaliana* roots.** Typical fluorescence (at λ_exc_ 365 and λ_em_ 460 nm) and absorbance (at 320 nm) chromatograms for root and growth media extracts from plants grown as described in Fourcroy et al. (2014): plants were pre-grown for 29 days in the presence of 45 μM Fe (III)-EDTA at pH 5.5, and then grown for 7 days in a medium with 0 (-Fe) or 45 μM Fe (III)-EDTA (+Fe) (the pH was not readjusted to 5.5, with the final pH being *c.* 7.0 in all pots). Chromatograms were obtained using Elution program 1. The encircled numbers above each peak correspond to the phenolic compounds listed in **Table 1**. RU, relative units, AU, absorbance units, and RT, retention time.

This is illustrated by the absorbance chromatograms of *A. thaliana* root extracts and growth media at 320 nm, which show many additional peaks to those found in fluorescence chromatograms obtained with the same samples (**Figure 3**). Each of the peaks in the chromatogram may contain one or more compounds (either fluorescent and/or non-fluorescent; see sections below for identification). In the control root extracts, fluorescence chromatograms showed only two peaks at approximately 10 and 15 min, whereas the absorbance chromatograms show several small peaks at two retention time (RT) ranges, 9–16 and 19–24 min, as well as a large peak at approximately 18 min (**Figure 3**). In the root extracts from Fe-deficient plants, increases were found in fluorescence in the area of the 15 min peak and in absorbance in the 18 min peak. In the control nutrient solution, the fluorescence chromatogram showed peaks at 10, 15, and 19 min, whereas the absorbance chromatogram showed peaks at 18 and 19 min (**Figure 3**). Iron deficiency caused large increases in the areas of all these peaks, with further absorbance ones appearing at 13, 14, 15, 16, and 17 min. This shows that Fe deficiency induces the synthesis, root accumulation and secretion to the growth media not only of fluorescent coumarins, as described by Fourcroy et al. (2014) and Schmid et al. (2014), but also of a number of previously unreported non-fluorescent phenolic compounds.

### Identification of Phenolic Compounds Induced by Fe Deficiency as Coumarins, Coumarin Precursors and Coumarinolignans

To identify the compounds found in the *A. thaliana* root extracts and growth media, samples were analyzed using four different HPLC-UV/VIS/ESI-MS(TOF) protocols, including two Elution programs (1 and 2; Supplementary Table S2) and two electrospray (ESI) ionization modes (positive and negative). The newly designed Elution program 2 led to a better separation of phenolic compounds than that obtained with the original Elution program 1 used in Fourcroy et al. (2014). With the new elution program, RTs for a selected set of phenolics standards ranged from 8.4 (for esculin, the glucoside form of the coumarin esculetin) to 51.7 min (for the flavone apigenin) (Supplementary Figures S1 and S2). These HPLC/ESI-MS(TOF) analyses provided highly accurate (error below 5 ppm) measurements of the mass-to-charge (*m/z*) ratio of the detected ions, therefore allowing for accurate elemental formulae assignments (Bristow, 2006).

Raw MS(TOF) datasets (time, *m/z* and ion intensity) from the root extracts and nutrient solutions from Fe-deficient and Fe-sufficient plants were first analyzed with the DISSECT algorithm (Data Analysis 4.0; Bruker) to obtain mass spectral features attributable to individual compounds. From a total of approximately 180 possible mass spectral features analyzed per run and sample, only 18 complied with the following two requirements: (i) occurring at chromatographic RTs where absorbance at 320 nm was observed, and (ii) showing peak area increases (or appearing) with Fe-deficiency. Then, associated ions coming from adducts (with salts or solvents), dimers and trimers were discarded (with some exceptions, see below), and the ion chromatograms of all major remaining ions (including non-fragmented ones as well as fragment ions produced in the ESI source) were extracted with a precision of ±0.02 *m/z*. From these, we selected major ions showing large changes in peak areas in response to Fe deficiency, without considering fragments and minor ions. The localization in the chromatograms of the 18 selected compounds is depicted in **Figure 3**, and the RT, exact *m/z* and assigned elemental formulae are shown in **Table 1**. These 18 compounds were never detected in nutrient solutions of pots without plants, and include some coumarins already known to occur and others not previously reported, as explained in detail below.

**Table 1:** Phenolic compounds secreted and accumulated by *Arabidopsis thaliana* roots in response to Fe deficiency: retention times (RT), exact mass-to-charge ratios (*m/z*), molecular formulae and error *m/z* (in ppm).

Compound #	RT (min) program 1	RT (min) program 2	Measured *m/z*	Molecular formula	Calculated *m/z*	Error *m/z* (ppm)	Annotation
*1*	9.8	10.3	355.1028	C_16_H_19_O_9_^+^ C_16_H_17_O_9_^-^	355.1024	1.1 2.8	7-hydroxy-6-methoxycoumarin hexoside (scopolin, scopoletin hexoside)
			353.0877		353.0867		
*2*	10.0	10.6	357.1182	C_16_H_21_O_9_^+^	357.1180	0.6	Ferulic acid hexoside
			355.1030	C_16_H_19_O_9_^-^	355.1024	1.7	
*3*	10.4	12.3	363.1055	C_16_H_20_O_8_Na^+^	363.1050	1.4	Coniferyl aldehyde hexoside
			339.1079	C_16_H_19_O_8_^-^	339.1074	-1.5	
*4*	11.3	13.0	371.0975	C_16_H_19_O_10_^+^ C_16_H_17_O_10_^-^	371.0973	0.5 3.0	7,8-dihydroxy-6-methoxycoumarin hexoside (fraxetin hexoside)
			369.0827		369.0816		
*5*	12.1	14.7	407.0949	C_17_H_20_O_10_Na^+^ C_17_H_19_O_10_^-^	407.0949	0.0 5.0	7-hydroxy-6,8-dimethoxycoumarin hexoside (isofraxidin hexoside)
			383.0992		383.0973		
*6*	12.3	14.9	409.0893	C_17_H_22_O_9_K^+^ C_17_H_21_O_9_^-^	409.0895	-0.5 3.8	Sinapyl aldehyde hexoside
			369.1194		369.1180		
*7*	13.0	16.4	209.0446	C_10_H_9_O_5_^+^ C_10_H_7_O_5_^-^	209.0445	0.5 -2.9	7,8-dihydroxy-6-methoxycoumarin (fraxetin)
			207.0282		207.0288		
*8*	14.5	20.0	193.0502	C_10_H_9_O_4_^+^ C_10_H_7_O_4_^-^	193.0495	3.6 1.0	7-hydroxy-6-methoxycoumarin (scopoletin)
			191.0341		191.0339		
*9*	14.8	21.6	223.0604	C_11_H_11_O_5_^+^ C_11_H_9_O_5_^-^	223.0601	1.3 -1.4	7-hydroxy-6,8-dimethoxycoumarin (isofraxidin)
			221.0442		221.0445		
*10*	15.6	23.0	195.0649	C_10_H_11_O_4_^+^ C_10_H_9_O_4_^-^	195.0652	-1.5 4.7	Ferulic acid
			193.0504		193.0495		
*11*	15.6	23.8	223.0604	C_11_H_11_O_5_^+^ C_11_H_9_O_5_^-^	223.0601	1.3 -1.4	6-hydroxy-5,7-dimethoxycoumarin (fraxinol)
			221.0442		221.0445		
*12*	16.1	24.6	179.0708	C_10_H_11_O_3_^+^ C_10_H_9_O_3_^-^	179.0703	2.7 2.8	Conyferyl aldehyde
			177.0551		177.0546		
*13*	16.5	25.1	209.0809	C_11_H_13_O_4_^+^ C_11_H_11_O_4_^-^	209.0808	0.5 3.9	Sinapyl aldehyde
			207.0660		207.0652		
*14*	16.5	30.7	403.1018	C_20_H_19_O_9_^+^ C_20_H_17_O_9_^-^	403.1024	-1.5 2.5	5′-hydroxycleomiscosins A and/or B
			401.0877		401.0867		
*15*	18.0	35.5	417.1175	C_21_H_21_O_9_^+^ C_21_H_19_O_9_^-^	417.1180	-1.2 -0.5	Cleomiscosin D
			415.1022		415.1024		
*16*	18.5	37.0	417.1173	C_21_H_21_O_9_^+^ C_21_H_19_O_9_^-^	417.1180	-1.7 -0.5	Cleomiscosin C
			415.1022		415.1024		
*17*	18.5	37.0	387.1073	C_20_H_19_O_8_^+^ C_20_H_17_O_8_^-^	387.1074	-0.3 3.1	Cleomiscosin B
			385.0930		385.0918		
*18*	19.0	38.6	387.1073	C_20_H_19_O_8_^+^ C_20_H_17_O_8_^-^	387.1074	-0.2 1.0	Cleomiscosin A
			385.0922		385.0918		

*The *m/z* ratios for [M+H]^+^ and [M-H]^-^ were determined from the HPLC/ESI-MS(TOF) data obtained in positive and negative mode, respectively. For compounds *3, 5*, and *6* in positive mode, the *m/z* shown are those measured for the Na ([M+Na]^+^) or K ([M+K]^+^) adducts, because they were more intense than those for [M+H]^+^. Common names for coumarins are also indicated in brackets.*

#### Coumarins and Related Compounds Previously Reported in *A. thaliana* upon Fe-Deficiency

As expected, some compounds (five out of 18) have RTs and *m/z* values matching with those of coumarins previously found in roots and exudates from Fe-deficient *A. thaliana* plants (Fourcroy et al., 2014; Schmid et al., 2014; Schmidt et al., 2014). These include compounds *1, 7–9*, and *11* (**Figure 3**; **Table 1**), and were assigned to scopoletin hexoside, fraxetin, scopoletin, isofraxidin and fraxinol (an isofraxidin isomer), respectively (Supplementary Table S1). These annotations were further confirmed using the RT and *m/z* values of standards (**Table 1** vs. **Table 2**). A sixth compound, *2*, was assigned to ferulic acid hexoside based on the presence of a major ion at *m/z* 195.0656 in its positive MS(TOF) spectrum, which is consistent with the elemental formula of ferulic acid [M+H]^+^ ion (**Table 2**) and with the neutral loss of a hexosyl moiety (162.0528 Da, C_6_H_10_O_5_) from the [M+H]^+^ ion (with an absolute error of 1.2 ppm). We could not confirm the identity using a ferulic acid hexoside standard because to the best of our knowledge no such standard is commercially available.

**Table 2:** Phenolic compound standards used for identification purposes: retention times (RT), exact mass-to-charge ratios (*m/z*), molecular formulae and error *m/z* (in ppm).

Name	RT (min) program 2	Measured *m/z*	Molecular formula	Calculated *m/z*	Error *m/z* (ppm)	ESI-MS^n^ *m/z* (Relative intensity, in %)
7-hydroxy-6-methoxycoumarin 7-glucoside (scopolin, scopoletin 7-*O*-glucoside)	10.3	355.1021	C_16_H_19_O_9_^+^	355.1024	-0.8	MS^2^ [355]: 337 (11), 245 (3), **193 (100)**, 149 (1), 165 (1), 133 (12), 105 (5) MS^3^ [355→193]: 178 (16), 165 (21), 149 (11), 137 (6), **133 (100)**
		353.0876	C_16_H_17_O_9_^-^	353.0867	2.5	MS^2^ [353]:**191 (100)**, 176 (9) MS^3^ [353→191]: **176 (100)**
7,8-dihydroxy-6-methoxycoumarin 8-glucoside (fraxin)	13.0	371.0956	C_16_H_19_O_10_^+^	371.0973	-4.6	MS^2^ [371]: 368 (11), 362 (13), 357 (12), 355 (66), 353 (35), 340 (13), 327 (23), 326 (25), 325 (195), 309 (15), 300 (17), 288 (10), 269 (19), 268 (11), 265 (11), 262 (14), 261 (17), 221 (12), **209 (100)**, 187 (19), 177 (14), 170 (19), 156 (15), 133 (24) MS^3^ [371→209]: **194 (100)**
		369.0825	C_16_H_17_O_10_^-^	369.0816	2.4	MS^2^ [369]: **207 (100)**, 192 (20) MS^3^ [369→207]: **192 (100)**, 163 (0.2)
7,8-dihydroxy-6-methoxycoumarin (fraxetin)	16.4	209.0444	C_10_H_9_O_5_^+^	209.0445	-0.5	MS^2^ [209]: 194 (31), 181 (52), 177 (15), 165 (7), 163 (80), 153(9), **149 (100)**, 135 (13), 107 (18)
		207.0291	C_10_H_7_O_5_^-^	207.0288	1.4	MS^2^ [207]: **192 (100)**, 163 (0.3)
7-hydroxy-6-methoxycoumarin (scopoletin)	20.0	193.0494	C_10_H_9_O_4_^+^	193.0495	-0.5	MS^2^ [193]: 178 (8), 165 (31), 149 (12), 137 (12), **133 (100)**, 117 (2), 105 (3), 89 (3), 63 (6)
		191.0346	C_10_H_7_O_4_^-^	191.0339	3.7	MS^2^ [191]: **176 (100)**, 148 (0.4)
7-hydroxy-6,8-dimethoxycoumarin (isofraxidin)	21.6	223.0594	C_11_H_11_O_5_^+^	223.0601	-3.1	MS^2^ [223]: **208 (100)**, 207 (7), 195 (14), 191 (8), 190 (49), 179 (7), 163 (72), 162 (6), 135 (19) 107 (45)
		221.0443	C_11_H_9_O_5_^-^	221.0445	-0.9	MS^2^ [221]: **206 (100)**, 209 (0.5), 191 (5), 162 (0.8)
Ferulic acid	23.0	195.0657	C_10_H_11_O_4_^+^	195.0652	2.6	MS^2^ [195]: **177 (100)**, 153 (4), 145 (3)
		193.0504	C_10_H_9_O_4_^-^	193.0495	4.7	MS^2^ [193]: 178 (70), **149 (100)**, 139 (80)
6-hydroxy-5,7-dimethoxycoumarin (fraxinol)	23.8	223.0594	C_11_H_11_O_5_^+^	223.0601	-3.1	MS^2^ [223]: **208 (100)**, 195 (11), 190 (40), 179 (6), 163 (54), 135 (19), 107 (39), 91 (4)
		221.0440	C_11_H_9_O_5_^-^	221.0444	-1.8	MS^2^ [221]: **206 (100)**, 191 (5), 209 (0.5), 162 (0.2)
Coniferyl aldehyde	24.6	179.0706	C_10_H_11_O_3_^+^	179.0703	1.7	MS^2^ [179]: **161 (100)**, 147 (97), 133 (18), 119 (7), 105 (10)
		177.0554	C_10_H_9_O_3_^-^	177.0546	4.5	MS^2^ [177]: **162 (100)**, 163 (1), 158 (0.3)
Sinapyl aldehyde	25.1	209.0810	C_11_H_13_O_4_^+^	209.0808	1.0	MS^2^ [209]: 191 (47), 181 (10), **177 (100)**, 153 (7), 149 (20), 145 (15), 131 (12), 121 (17), 103 (5)
		207.0662	C_11_H_11_O_4_^-^	207.0652	4.8	MS^2^ [207]: **192 (100)**, 191 (0.3), 177 (2), 147 (0.2), 133 (0.2)

*The *m/z* ratios of parent and fragment ions were determined from the data in the HPLC/ESI-MS(TOF) and HPLC/ESI-MS(ion trap) chromatograms, respectively, working in both positive and negative mode. Common names for coumarins and their glucosides are indicated in brackets. The parent ion *m/z* ratios correspond to [M+H]^+^ and [M-H]^-^. The major ion of the MS^2^ and MS^3^ spectra is indicated in bold.*

The remaining 12 compounds were subjected to further MS-based analyses to obtain structural information. First, low resolution HPLC/ESI-MS(ion trap) analyses were carried out, including MS^2^ and/or MS^3^ experiments with the [M+H]^+^ or [M-H]^-^ ions.

#### Coumarins and Coumarin-Precursor Hexosides Not Previously Reported in *Arabidopsis* upon Fe-Deficiency

Three of the compounds (*10, 12*, and *13*) were identified as ferulic acid, coniferyl aldehyde and sinapyl aldehyde (three phenylpropanoid precursors; **Figure 1B**), respectively, by comparing the MS spectra of the analytes and those of standards: there was a good match of the RT values and exact *m/z* ratios of the [M+H]^+^ and [M-H]^-^ ions (**Tables 1** and **2**) as well as of the MS^2^ spectra of the [M+H]^+^ ions (**Tables 2** and **3**).

**Table 3:** MS/MS data for some of the compounds secreted and accumulated by *Arabidopsis thaliana* roots in response to Fe deficiency: *m/z* ratios of the fragment ions and their relative intensity.

*Compound #*	Annotation	Parent ion *m/z*	Ion type	ESI-MS^n^ *m/z* (Relative intensity, in %)
*3*	Coniferylaldehyde hexoside	339.1	[M-H]^-^	MS^2^ [339]: 295 (6), 275 (8), 250 (6), 249 (3), 188 (3), **177 (100)**, 162 (3) MS^3^ [339→177]: **162 (100)**
*4*	7,8-dihydroxy-6-methoxycoumarin hexoside (fraxetin hexoside)	369.1	[M-H]^-^	MS^2^ [369]: 325 (7), 323 (5), 223 (11), 215 (8), **207 (100)**, 193 (5), 192 (20) MS^3^ [369→207]: **192 (100)**
*5*	7-hydroxy-6,8-dimethoxycoumarin hexoside (isofraxidin hexoside)	383.1	[M-H]^-^	MS^2^ [383]: 365 (13), 347 (24), 341 (12), 339 (10), 337 (22), 323 (24), 322 (18), 303 (14), 270 (20), 268 (25), 266 (18), 252 (9), 251 (30), **221 (100)**, 215 (38), 207 (7), 206 (11), 203 (11), 199 (15), 187 (8), 177 (20), 173(8), 156 (11), 131 (17), 129 (30), 125 (6), 114 (24) MS^3^ [383→221]: **206 (100)**
*6*	Sinapyl aldehyde hexoside	369.1	[M-H]^-^	MS^2^ [369]: 351 (33), 325 (11), 289 (10), 254 (5), 253 (6), 246 (11), 245 (8), 239 (9), 237 (11), 217 (6), **207 (100)**, 192 (18), 159 (11), 128 (10) MS^3^ [369→207]: **192 (100)**
*10*	Ferulic acid	193.1	[M-H]^-^	MS^2^ [193]: 178 (70), **149 (100)**, 134 (72)
*12*	Coniferyl aldehyde	179.1	[M+H]^+^	MS^2^ [179]: 161 (86), **147 (100)**, 133 (17), 119 (10), 105 (8)
*13*	Sinapyl aldehyde	209.1	[M+H]^+^	MS^2^ [209]: 191 (41), 181 (17), **177 (100)**, 149 (22), 145 (13), 131 (5), 121 (18)

*Numbers in italics (Compound #) refer to the labels used for each compound in **Table 1**. All data were taken from the HPLC-ESI-MS/MS(ion trap) analysis. The major ion of the MS^2^ and MS^3^ spectra is also indicated in bold.*

Four more compounds (*3–6*) were first confirmed to be hexoside-type compounds from the RT, exact *m/z* values and MS^2^ spectra of the [M-H]^-^ ions. The RT values of these compounds (12.3–14.9 min) were close to those of known coumarin glucosides (10.3 and 13.0 min for scopolin and fraxin, respectively), and lower than those of coumarin aglycones (16.4–25.1 min for fraxetin, scopoletin, isofraxidin and fraxinol), phenylpropanoids (e.g., 23.0 and 25.1 min for ferulic acid and sinapyl aldehyde), and glycoside and aglycone forms of other phenolics (e.g., 27–52 min for flavonoids, stilbenes and lignans) (Supplementary Figures S1 and S2). Therefore, the RTs indicate that compounds *3–6* are likely to be polar (i.e., hexoside) forms of coumarins and/or phenylpropanoids. Furthermore, in the MS(TOF) spectra, ions (positive/negative) at *m/z* 179.0707/177.0544, 209.0450/207.0289, 223.0600/221.0447 and 209.0801/207.0648 for *3, 4, 5*, and *6*, respectively, were consistent with the loss of a hexosyl moiety (162.05 Da) from their corresponding [M+H]^+^/[M-H]^-^ ions (see *m/z* values in **Table 1**). This was confirmed using the low resolution MS^2^ spectra obtained with the ion trap: major fragment ions (100% relative intensity at *m/z* 177, 207, 221 and 207 in the MS^2^ spectra of *3*–*6*, respectively; **Table 3**) corresponded to the [M-H]^-^ ions (*m/z* 339, 369, 383 and 369 for *3, 4, 5*, and *6*, respectively) after a mass loss of 162 Da. The same mass loss was also observed in the MS^2^ spectra of authenticated standards of the coumarin glucosides scopolin and fraxin described above, with major ions at *m/z* 193/191 (scopolin) and 209/207 (fraxin), corresponding with the *m*/*z* of their aglycones, scopoletin and fraxetin, respectively (**Table 2**). The rest of ions in the MS^2^ spectra of compounds *3–6*, scopolin and fraxin showed significantly lower relative intensities (<40%), indicating the hexosyl loss is favored.

The aglycon moieties of compounds *3–6* were identified taking advantage of having the dehexosylated ions in the MS(TOF) spectra and also carrying out low resolution MS^3^ experiments on the ion trap. First, from the positive and negative MS(TOF) spectra, the *m/z* values for dehexosylated ions (see above) of *3, 4, 5*, and *6* were assigned to the elemental formulae C_10_H_10_O_3_, C_10_H_8_O_5_, C_11_H_10_O_5_ and C_11_H_12_O_4_, respectively (with absolute errors <4 ppm). Two of these elemental formulae, C_10_H_10_O_3_ and C_11_H_12_O_4_, were consistent with coniferyl and sinapyl aldehydes, involved in coumarin synthesis (Kai et al., 2008) (**Table 2**), whereas the other two, C_10_H_8_O_5_ and C_11_H_10_O_5_, were consistent with two coumarins already identified in the samples (compounds *7* and *9*, respectively) (**Table 1**). Finally, compounds *3–6* were confirmed as the hexoside forms of coniferyl aldehyde, fraxetin, isofraxidin and sinapyl aldehyde, respectively (**Table 1**) from the good fit between the MS^3^ ion trap spectra of *3-6* (339→177, 369→207, 383→221 and 369→207, respectively) (**Table 3**) and the MS^2^ spectra of the corresponding aglycone standards (**Table 2**).

#### Coumarinolignans: Newly Identified Compounds Synthesized in Response to Fe-Deficiency

The last five compounds (*14*–*18* in **Table 1**) are very hydrophobic, since they elute later (RTs 31–39 min) than compounds *1–13* (RTs 10–25 min), and have *m/z* values supporting elemental formulae with a high number of C atoms (20–21 vs. 10–17 for compounds *1–13*). In fact, the RTs of *14*–*18* are in line with those of phenolics bearing either C_15_ (C_6_-C_3_-C_6_; as in flavonoids and stilbens) or C_18_ (C_6_-C_3_-C_3_-C_6_; as in lignans) skeletons (27–52 min; Supplementary Figures S1 and S2), whereas compounds *7–13* (coumarins and phenylpropanoids) share a C_9_ (C_3_–C_6_) skeleton and compounds *1*–*6* (hexose conjugates of *7*–*13*) share a C_15_ (C_3_-C_6_-C_6_) skeleton (**Table 1**).

**FIGURE 4:**
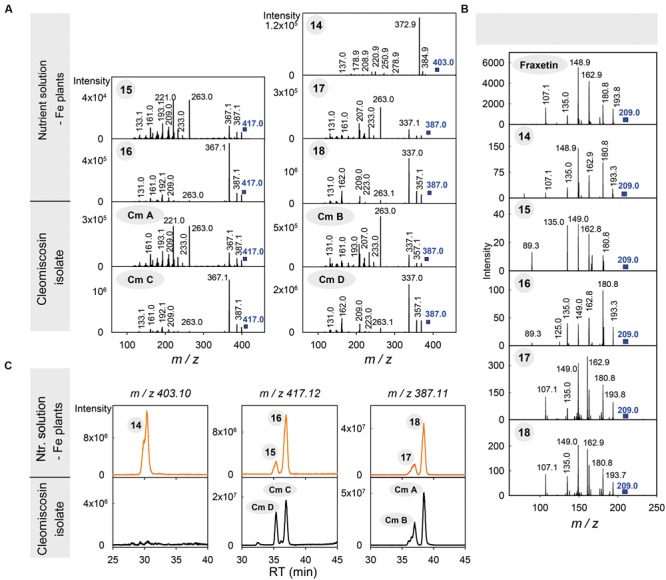
**Identification of compounds *14*–*18*, produced by Fe-deficient *Arabidopsis thaliana* roots, as coumarinolignans derived from fraxetin. (A)** MS^2^ spectra of compounds *14*–*18* and the cleomiscosins A (Cm A), B (Cm B), C (Cm C) and D (Cm D) isolated from *Cleome viscosa* seeds. **(B)** MS^2^ spectra of fraxetin and MS^3^ spectra of *m/z* 209 ion from the corresponding [M+H]^+^ ions of compounds *14*–*18*. Spectra were obtained from the HPLC/ESI-MS(ion trap) analyses of growth media extracts from Fe-deficient plants and a cleomiscosin isolate. **(C)** Typical HPLC-ESI-MS(TOF) chromatograms for growth media extracts from Fe-deficient plants and for the cleomiscosin isolate, extracted at *m/z* 403.10, 417.12 and 387.11 and with a precision of ± 0.02 *m/z* units. The encircled numbers in the spectra and above each chromatographic peak correspond to the phenolic compounds listed in **Table 1**.

The MS(TOF) spectra show that compounds *15*–*18* are two pairs of isomers, with elemental formulae C_21_H_20_O_9_ for *15*–*16* and C_20_H_18_O_8_ for *17*–*18*, with the difference between formulae being consistent with a single methoxy (-OCH_3_) group. The elemental formula of compound *14*, C_20_H_18_O_9_, is consistent with the addition of both a hydroxyl (-OH) group to *17*–*18* or the addition of a methyl (-CH_3_) group to *15*–*16*. The presence of these structural differences are common among phenolics, since part of the phenylpropanoid biosynthesis proceeds *via* a series of ring hydroxylations and *O*-methylations. The low resolution MS^2^ spectra from *14* to *18* (**Figure 4A**) indicate that these five compounds have highly related chemical structures: (i) the spectra of *15*–*16* show the same ions with only some differences in their relative intensity, and the same was also observed for *17*–*18*; (ii) most of the ions in the *15*–*18* spectra were either common (*m/z* 263, 233, 209, 161) or consistent with common mass losses from the [M+H]^+^ ion (e.g., *m/z* 367 and 337 in the *15*–*16* and *17*–*18* MS^2^ spectra, corresponding to a mass loss of 50 Da; Supplementary Table S8), and (iii) the spectrum of *14* also has some of these features, including an ion at *m/z* 209 and a mass loss of 30 Da from the [M+H]^+^ ion (Supplementary Table S8). When the MS^2^ spectra of *14*–*18* were obtained on a high resolution Q-TOF mass analyzer, which allows for an accurate mass determination of fragment ions, all spectra showed a common fragment ion at *m/z* 209.0435, consistent with the elemental formula C_10_H_9_O_5_^+^ (with an error of -4.7 ppm) (Supplementary Figure S3) of the dihydroxymethoxycoumarin fraxetin (compound *7*). The presence of a fraxetin moiety in compounds *14*–*18* was further confirmed by their MS^3^ spectra (403→209, 417→209, 417→209, 387→209 and 387→209 for *14, 15, 16, 17* and *18*, respectively; **Figure 4B**), which match perfectly with the fraxetin MS^2^ spectrum.

Among the plant-derived fraxetin derivatives known so far (Begum et al., 2010; Zhang et al., 2014), six coumarinolignans have elemental formulae consistent with those of compounds *14*–*18*, including cleomiscosins A, B, C (also known as aquillochin) and D, first isolated and identified in seeds of *Cleome viscosa* (a common weed of the *Capparidaceae* family), and 5′-hydroxycleomiscosins A (also known as 5′-demethylaquillochin) and B, first isolated from *Mallotus apelta* roots and *Eurycorymbus cavaleriei* twigs, respectively. Cleomiscosins C and D (regioisomers -also called constitutional isomers- arising from the fusion of fraxetin and the monolignol sinapyl alcohol through a dioxane bridge; **Figure 1C**) have a formula identical to that of *15*–*16* (C_21_H_20_O_9_), cleomiscosins A and B (regioisomers arising from the fusion of fraxetin and the monolignol coniferyl alcohol through a dioxane bridge; **Figure 1C**) have a formula identical to that of *17*-*18* (C_20_H_18_O_8_), whereas 5′-hydroxycleomiscosins A and B (regioisomers arising from the fusion of fraxetin and the monolignol hydroxyconiferyl alcohol, Cheng and Chen, 2000, **Figure 1C**), have a formula identical to that of compound *14* (C_20_H_18_O_9_). The structural differences among these coumarinolignans -corresponding to the monolignol moiety (**Figure 1B**)- are identical to those found among the elemental formulae of *14*–*18*: (i) a methoxy group differentiates coniferyl from sinapyl alcohols and the elemental formula of *17*–*18* from that of *15–16*; (ii) a hydroxyl group differentiates hydroxyconiferyl from coniferyl alcohols and the elemental formula of *14* from that of *17*–*18*; and (iii) a methyl group differentiates hydroxyconiferyl and sinapyl alcohols and the formula of *14* from those of *15*–*16*.

To confirm the identification of *15*–*18* as cleomiscosins, we isolated coumarinolignans from *C. viscosa* seeds. The seed isolate was analyzed by both HPLC-UV/VIS/ESI-MS(TOF) and HPLC/ESI-MS(ion trap) using Elution program 2 and positive ESI ionization. The HPLC/ESI-MS(TOF) chromatogram for *m/z* 417.12 ± 0.02, corresponding to the cleomiscosins C and D [M+H]^+^ ions, showed only two peaks, at 35.4 and 37.0 min, matching with the RTs of *15* and *16* (**Figure 4C**; **Table 1**). Similarly, the HPLC/ESI-MS(TOF) chromatogram for *m/z* 387.11 ± 0.02, corresponding to the cleomiscosins A and B [M+H]^+^ ions, showed only two peaks, at 37.0 and 38.4 min, matching with the RTs of *17*–*18* (**Figure 4C**; **Table 1**). Peaks were assigned to cleomiscosin isomers according to the elution order reported in the literature (Chattopadhyay et al., 2008; Kaur et al., 2010). These annotations were confirmed by the full match between the MS^2^ spectra of the cleomiscosins D, C, B, and A, and those of compounds *15, 16, 17* and *18*, respectively (**Figure 4C**). Compound *14* eluted at shorter times than the cleomiscosins (30.7 vs. 35.5–38.6 min), as expected from the structural differences between 5′-hydroxycleomiscosin A and B and cleomiscosins (see above). Furthermore, compound *14* shares elemental formula and the presence of a fraxetin moiety with 5′-hydroxycleomiscosins A and B, and its MS^2^ spectrum showed a loss of 18 Da from the [M+H]^+^ ion (**Figure 4B**; Supplementary Table S8), which was previously reported for 5′-hydroxycleomiscosin A (Cheng and Chen, 2000) but does not occur in cleomiscosins. Therefore, *14* was putatively annotated as 5′-hydroxycleomiscosin A and/or B (**Table 1**).

### Coumarin and Coumarinolignan Concentrations in Root Extracts

Quantification of phenolic compounds was carried out using the [M+H]^+^ and [M-hexoside+H]^+^ signals in the HPLC/ESI-MS(TOF). Coumarins and their hexosides were quantified using authenticated standards, whereas coumarinolignan concentrations were estimated using peak/area ratios relative to that of the IS lignan matairesinol (**Figure 1D**), because of the lack of commercially available authenticated standards.

**FIGURE 5:**
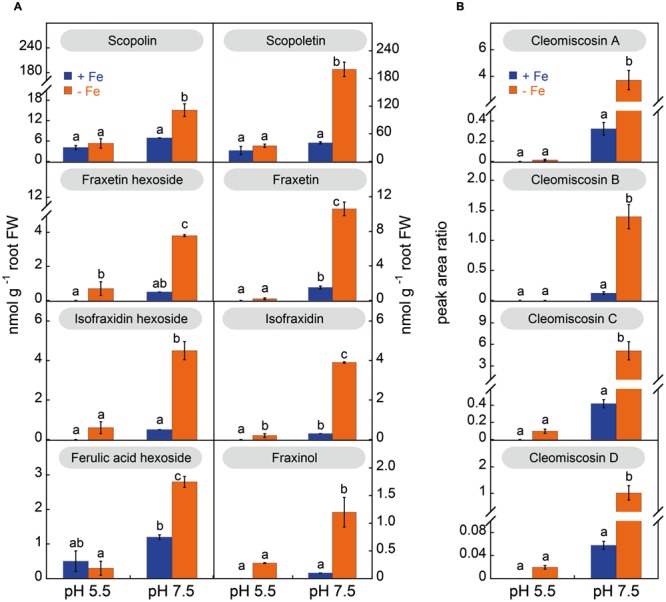
**Effects of Fe deficiency and high pH on the concentrations (in nmol g^-1^ root FW) of coumarins (A) and coumarinolignans (B) in *Arabidopsis thaliana* roots.** Plants were pre-grown as indicated in **Figure 2** and grown for 14 days with 0 (-Fe) or 20 μM Fe (+Fe) in nutrient solution buffered at pH 5.5 (with 5 mM MES-NaOH) or 7.5 (with 5 mM HEPES-NaOH). Ferulic acid hexoside was quantified as fraxin. The levels of the cleomiscosins are expressed in peak area ratio, relative to the lignan matairesinol used as internal standard. Data are means ± SE (*n* = 3–5). For each compound, significant differences among treatments (at *p* < 0.05) are marked with different letters above the columns.

The phenolic compound profiles in root extracts included coumarins and coumarinolignans, and were markedly dependent on the plant growth pH (**Figure 5**); no phenolics of the flavonoid and stilbene families were found. Under sufficient Fe supply, root extracts from plants grown at pH 5.5 had mainly scopoletin hexoside (scopolin) and its aglycone (scopoletin) as well as the coumarin precursor hexoside of ferulic acid. When Fe-sufficient plants were grown at pH 7.5, no significant changes were found for ferulic acid hexoside, scopolin, scopoletin and fraxetin and isofraxidin hexosides, and the coumarinolignans cleomiscosins A, B, C, and D, whereas other coumarins increased (including fraxetin and isofraxidin).

Iron deficiency changed markedly the coumarin/coumarinolignan profiles in root extracts (**Figure 5**). In plants grown at pH 5.5 the profiles were similar under Fe deficiency or sufficiency conditions, with moderate increases (not always significant) in fraxetin and isofraxidin hexosides and their aglycones (fraxetin, isofraxidin and fraxinol), as well as of the cleomiscosins A, B, C and D. However, in plants grown at pH 7.5 Fe deficiency caused a marked increase of all coumarin hexosides, their aglycones and all coumarinolignans. When compared to their concentration in Fe-sufficient plants at pH 7.5, the largest increase was 18-fold for cleomiscosin D, followed by 13-fold for isofraxidin, 12-fold for fraxinol and the cleomiscosins A, B, and C, 9-fold for the hexoside of isofraxidin, 7-fold for the hexoside of fraxetin and the aglycone fraxetin, 5-fold for scopoletin, and 2-fold for both scopolin and ferulic acid hexoside.

**FIGURE 6:**
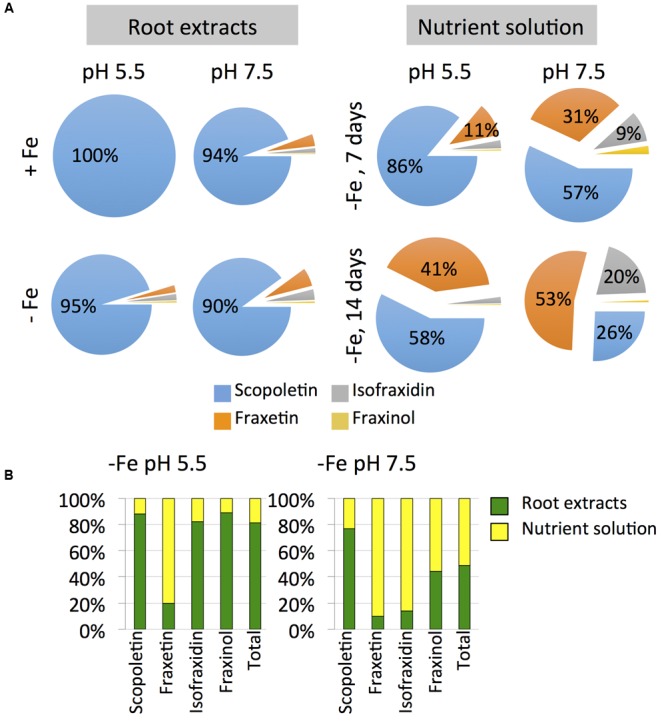
**Effects of Fe deficiency, high pH and/or time on the relative concentrations of coumarins (scopletin, fraxetin, isofraxidin, fraxinol and total coumarins) in root extracts and nutrient solution (A) and on the allocation of coumarins to the roots and the nutrient solutions of *Arabidopsis thaliana***(B)**.** Plants were pre-grown as indicated in **Figure 2** and grown for 7 or 14 days with 0 (-Fe) or 20 μM Fe (+Fe) in nutrient solution buffered at pH 5.5 (with 5 mM MES-NaOH) or 7.5 (with 5 mM HEPES-NaOH). Data are means of *n* = 3–5. The absolute values are shown in **Figures 5** and **7**.

The most abundant coumarin in root extracts, irrespective of the growth conditions, was scopoletin (**Figure 6A**). Summing up the two forms detected, the hexoside and aglycone, scopoletin was 90–100% of the total coumarins, depending on the root conditions, with the aglycone form being always predominant (85–93%) (Supplementary Figure S4B). In the case of fraxetin, the aglycone was also the predominant form (at least 73–76%) in root extracts from plants grown at pH 7.5, whereas in plants grown in absence of Fe at pH 5.5, only 24% of the total fraxetin occurred in the aglycone form. In the case of isofraxidin the hexoside form was predominant, with the aglycone accounting for 23–46% of the total depending on the growth conditions (Supplementary Figure S4B).

### Coumarin and Coumarinolignan Concentrations in the Nutrient Solution

**FIGURE 7:**
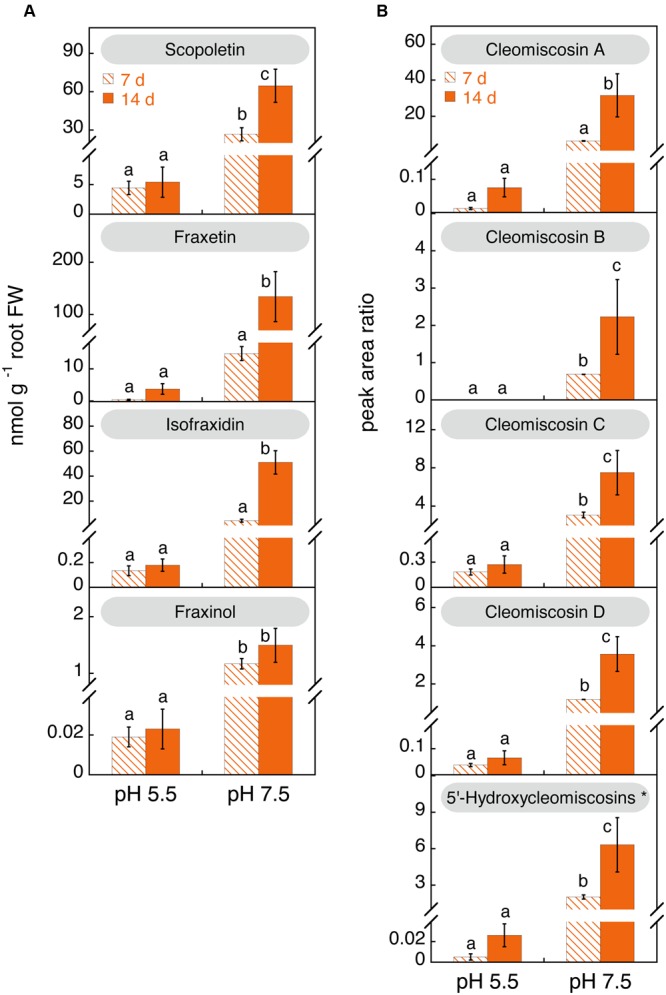
**Effects of time of Fe deficiency and high pH treatments on the concentrations (in nmol g^-1^ root FW) of coumarins (A) and coumarinolignans **(B)** in the nutrient solution of iron (Fe)-deficient *Arabidopsis thaliana*.** Plants were pre-grown as indicated in **Figure 2** and grown for 7 or 14 days with 0 μM Fe in nutrient solution buffered at pH 5.5 (with 5 mM MES-NaOH) or 7.5 (with 5 mM HEPES-NaOH). The levels of the cleomiscosins are expressed in peak area ratio, relative to the lignan matairesinol used as internal standard. Data are means ± SE (*n* = 3–5). For each compound, significant differences among treatments (at *p* < 0.05) are marked with different letters above the columns. ^∗^5′-Hydroxycleomiscosins A and/or B should be considered since separation of these isomer compounds might have not been achieved.

The concentrations of coumarins and coumarinolignans were determined in the nutrient solution of Fe-deficient plants after 7 and 14 days after imposing Fe deficiency (nutrient solutions were renewed on day 7) (**Figure 7**). No determinations could be made in nutrient solutions of Fe-sufficient plants due to the presence of Fe(III)-EDDHA, which causes the overloading of C_18_ materials. Coumarin hexosides were only occasionally detected at trace levels (data not shown). When plants were grown at pH 5.5, the growth media at day 7 contained low concentrations of aglycones (scopoletin, fraxetin, isofraxidin, and fraxinol; **Figure 7**) and coumarinolignans (cleomiscosins A, C, and D as well as the putative 5′- hydroxycleomiscosin; **Figure 7**). After 14 days of Fe deficiency no significant changes were observed. In contrast, when plants were grown at pH 7.5, the concentration of coumarins and coumarinolignans in the nutrient solution were much higher than that found in the culture medium of plant grown at pH 5.5 (**Figure 7**). When compared to the concentrations found with Fe-deficient plants at pH 5.5, increases were large for scopoletin (6- and 12-fold at days 7 and 14, respectively) and very large for the rest of phenolics (in the range from 17- to 537-fold). In addition, when Fe-deficient plants were grown at pH 7.5, the concentrations of coumarins (with the exception of fraxinol) and coumarinolignans in the nutrient solution increased with time. When compared to the concentrations at day 7, increases at d 14 were 12-fold for isofraxidin, 9-fold for fraxetin, 5-fold for cleomiscosin A, 3-fold for 5′-hydroxycleomiscosins and the cleomiscosins B and D, and 2-fold for scopoletin and cleomiscosin C.

Scopoletin was the predominant coumarin only at pH 5.5 after 7 days of Fe deficiency (86% of the total coumarins), whereas at 14 days scopoletin and fraxetin accounted for 58 and 41% of the total, respectively (**Figure 6A**). At pH 7.5 scopoletin and fraxetin were the major coumarins at day 7 (57 and 31%, respectively), whereas at d 14 scopoletin, fraxetin and isofraxidin accounted for 26, 53, and 20% of the total, respectively.

### Allocation of Coumarins to the Roots and the Nutrient Solutions

The allocation of coumarins produced by Fe-deficient plants was affected by the growth media pH. In plants grown at pH 5.5, only 19% of the total amount of coumarins was allocated to the nutrient solution, whereas for plants grown at pH 7.5 coumarins were allocated equally between nutrient solutions (51% of the total per plant) and roots (49%) (**Figure 6B**). Fraxetin was preferentially allocated to the nutrient solution at both pH values, whereas isofraxidin and fraxinol did only so at pH 7.5.

### Mobilization of Fe from Fe(III)-Oxide Promoted by Coumarins

**FIGURE 8:**
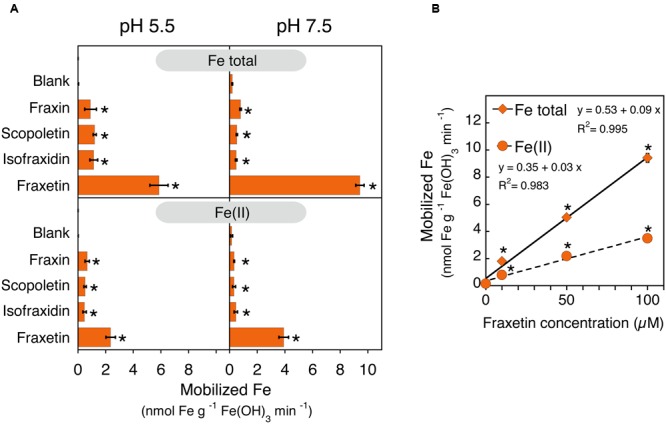
**Iron mobilization from an scarcely soluble Fe(III)-oxide as affected by coumarins. (A)** Structure-activity relationship of coumarins on Fe mobilization activity. The assays consisted in the incubation of 10 mg of Fe(III)-oxide with a solution of 0 (blank) or 100 μM of the indicated coumarins and 600 μM BPDS at two different pH values, 5.5 and 7.5. Total Fe and Fe(II)-(BPDS)_3_ in solution were determined by ICP-MS and spectrophotometry, respectively. **(B)** Effects of the fraxetin concentration on the Fe mobilization activity at pH 7.5. Scatter plot of the concentration of fraxetin vs. the total Fe mobilized and the Fe(II), with linear regression lines in black and their corresponding equations. In all cases **(A,B)**, data are means ± SE (*n* = 3–12) and asterisks denote a statistically significant difference between blank and a coumarin-containing assay medium as determined by Student’s *t*- test (*p* < 0.05).

In order to understand the role that coumarins could play in Fe plant nutrition, their ability to mobilize Fe from Fe(III)-oxide was measured in *in vitro* incubation assays. The experiments were carried out with a poorly crystaline Fe(III)-oxide and 1.5 ml of an assay medium containing 0 (blank) or 100 μM of coumarin and buffered at pH 5.5 or 7.5. Three out of the four coumarins assayed (scopoletin, isofraxidin and fraxin) have a catechol moiety capped *via* hydroxyl group methylation or hydroxyl group glucosylation, whereas the fourth coumarin, fraxetin, bears an available catechol moiety (see structures in **Figure 1A**). Coumarolignans could not be used in these experiments because of the lack of commercial authenticated standards. Assays were run in the presence of the Fe(II) trapping agent BPDS to monitor the reductive dissolution of Fe(III)-oxide, and the concentration of Fe(II)-BPDS_3_ was termed Fe(II). The overall mobilization of Fe was assessed by determining the total Fe in solution using ICP-MS (**Figure 8**). The Fe mobilized by the buffer solutions (blanks) was on the average 0.2 nmol Fe g^-1^ Fe(III)-oxide min^-1^. When the assay medium contained the non-catechol coumarins fraxin, scopoletin and isofraxidin, the total Fe mobilized was in the range 0.9–1.2 nmol Fe g^-1^ Fe(III)-oxide min^-1^ (depending on the coumarins and the assay pH) and statistically significant differences were found when compared to the blank (**Figure 8A**). However, when the assay medium contained the catechol coumarin fraxetin, the amounts of Fe mobilized (5.8 and 9.4 nmol Fe g^-1^ Fe(III)-oxide min^-1^ for the assays at pH 5.5 and pH 7.5, respectively) were significantly higher than the rest (**Figure 8A**). Furthermore, the total mobilization of Fe promoted by fraxetin at pH 7.5 increased linearly when the concentration of fraxetin increased from 10 to 100 μM. A relevant fraction (40–44%) of the mobilized Fe was trapped by BPDS and this fraction also increased linearly when the concentration of fraxetin increased from 10 to 100 μM (**Figure 8B**).

## Discussion

*Arabidopsis thaliana* plants produce and secrete an array of phenolics in response to Fe deficiency when the pH of the nutrient solution is high. Phenolics found in this study include several coumarinolignans not previously reported in *A. thaliana* (cleomiscosins A, B, C, and D and the 5′-hydroxycleomiscosins A and/or B), as well as other previously reported coumarins (scopoletin, fraxetin, isofraxidin and fraxinol) and some coumarin precursors (ferulic acid and coniferyl and sinapyl aldehydes). The identification of all these phenolic compounds was achieved through an integrative interpretation of analytical data, including exact molecular mass-to-charge ratios (*m/z*), low and high-resolution MS^n^ spectra, chromatographic RTs and fluorescence/UV-VIS data. Furthermore, we report here for the first time on the quantification of all identified coumarins, revealing that Fe deficiency mainly induced the root accumulation and exudation of the non-catechol coumarin scopoletin and the catechol coumarin fraxetin, with the exudation of fraxetin being more prominent when Fe chlorosis was intense. Also, we show for the first time that fraxetin, but not scopoletin, was effective to mobilize Fe from an scarcely soluble Fe(III)-oxide.

This is the first time cleomiscosins and 5′-hydroxycleomiscosins have been reported in *A. thaliana*. Cleomiscosins were found in both roots and nutrient solutions, whereas 5′-hydroxycleomiscosins were found only in nutrient solutions (**Figures 5B** and **7B**). All coumarinolignans found have a fraxetin moiety linked to different phenylpropanoid units (**Figure 1C**). Non-conventional lignans, including coumarinolignans and other hybrid ones, harbor a single phenylpropanoid unit, whereas conventional ones consist in phenylpropanoid dimers. The common coumarin moiety in the coumarinolignans found, fraxetin, has been consistently reported to increase with Fe deficiency in roots and growth media of *A. thaliana* (**Figures 5** and **7**; Fourcroy et al., 2014; Schmid et al., 2014; Schmidt et al., 2014). The phenylpropanoid units found are the primary lignin precursors coniferyl (in cleomiscosins A and B) and sinapyl alcohols (in cleomiscosins C and D), and the non-canonical monolignol 5-hydroxyconiferyl alcohol (in 5′-hydroxycleomiscosins A and B) (Begum et al., 2010) (**Figure 1C**). Previously, two other coumarinolignans, composed of esculetin and either coniferyl alcohol or sinapyl alcohol, were tentatively identified in *A. thaliana* root exudates (Strehmel et al., 2014). Until now, cleomiscosins have been only reported in seeds and stem wood and bark of various plant species, whereas 5′-hydroxycleomiscosins A and B were found in *Mallotus apelta* roots (Xu et al., 2008) and *Eurycorymbus cavaleriei* twigs (Ma et al., 2009), respectively. Cleomiscosin A has been reported in 22 plant species belonging to 12 families (e.g., Sapindaceae and Simaroubaceae), whereas cleomiscosins B, C, and D, although less common, have been found in 6–10 plant species belonging to 5–9 families (Begum et al., 2010).

Besides coumarinolignans, ferulic acid and other related metabolites were found to accumulate in roots of Fe-deficient *A. thaliana* plants when grown at high pH (**Table 1**; **Figure 5A**). This is consistent with Fe-deficient *A. thaliana* root transcriptomic (Rodríguez-Celma et al., 2013), proteomic (Lan et al., 2011) and metabolite data (Fourcroy et al., 2014): (i) ferulic acid can be converted to feruloyl-CoA by the action of 4-coumarate:CoA ligases (4CL1 and 4CL2), two enzymes that have been found to be robustly induced by Fe deficiency (Lan et al., 2011; Rodríguez-Celma et al., 2013), (ii) feruloyl-CoA is a key precursor in the biosynthesis of scopoletin (Kai et al., 2008), which accumulates in roots of Fe-deficient plants (**Figures 5A** and **7A**; Fourcroy et al., 2014; Schmid et al., 2014; Schmidt et al., 2014), and (iii) ferulic acid hexoside has been reported to occur in Fe-deficient roots (Fourcroy et al., 2014). Also, two other metabolites, coniferyl and sinapyl aldehydes, were occasionally found in Fe-deficient roots (in the aglycone and hexoside forms, **Tables 1** and **3**). Coniferyl aldehyde can either lead to scopoletin biosynthesis *via* oxidation to ferulic acid (Kai et al., 2008) or be reduced to coniferyl alcohol (Fraser and Chapple, 2011), a precursor of lignin and lignans (Barros et al., 2015), including cleomiscosins A and B. Sinapyl aldehyde is an intermediate metabolite in the synthesis of lignin and lignans such as cleomiscosins C and D (Barros et al., 2015), and may (assuming that isofraxidin synthesis is analogous to that of scopoletin, as proposed by Petersen et al., 1999) be a precursor of the coumarin isofraxidin, which accumulates consistently in Fe-deficient roots (**Figure 5A**).

Coumarins also accumulate in *A. thaliana* roots along with coumarinolignans and are secreted to the growth media in response to Fe deficiency, especially when pH was high. Four coumarins (scopoletin, fraxetin, isofraxidin and the isofraxidin isomer fraxinol) were found in both root extracts and nutrient solutions (**Tables 1** and **2**) confirming previous results (Fourcroy et al., 2014; Schmid et al., 2014; Schmidt et al., 2014) (Supplementary Table S1). We could identify fraxinol (annotated in a previous study as methoxyscopoletin; Fourcroy et al., 2014), using an authenticated standard. Aglycones and hexose conjugates of the four coumarins were found in roots (**Figure 5**; Supplementary Figure S4B), whereas only the aglycone forms were quantifiable in nutrient solutions, with hexoside forms being detected only occasionally and in low amounts (**Figure 7**). We did not detect three more coumarins, esculetin, isofraxetin and dihydroxyscopoletin, previously found as aglycones and/or glycoside forms by Schmid et al. (2014) and/or Schmidt et al. (2014) in roots or exudates of Fe-deficient *A. thaliana*. This could be due to differences in protocols for exudate collection and isolation of organic compounds from the growth/exudation media or plant growth conditions. In any case, from the published data it seems that the relative amount of these three coumarins was very low: in the only study where quantification of some coumarins was carried out, the amount of esculetin was 0.1% (roots) and <1% (exudates) when compared to those of scopoletin (Schmid et al., 2014). Assuming similar ratios in our study, the concentration of esculetin would be approximately 0.2–0.5 nmol g^-1^ root FW in roots and nutrient solutions, respectively, values still lower than those of fraxinol, the least abundant of the coumarins detected in this work (**Figures 5** and **7**). Regarding the other two coumarins not detected in this study, isofraxetin and dihydroxyscopoletin, they were only detected in Schmid et al. (2014) and Schmidt et al. (2014), respectively, indicating that their occurrence in Fe-deficient plants is not consistent.

High pH induces by itself a certain Fe stress that results in the synthesis of phenolics in roots. The increase in the production of some phenolic compounds was already observed in Fe-sufficient plants grown at high pH (**Figure 5**; Supplementary Figure S4A), along with decreases in root and shoot Fe contents (**Figure 2C**) and increases in *FRO2* expression (**Figure 2D**), even when leaf Chl and biomass were not affected (**Figures 2A–C**). It was already known that high pH compromises the root Fe acquisition from Fe(III)-chelates, with FCR activities being much lower at pH 7.5 than at the optimal pH range of 5.0-5.5 (in *A. thaliana* and other species; Moog et al., 1995; Susín et al., 1996), and FCR rates are known to be especially low with highly stable chelates such as Fe(III)-EDDHA (Lucena, 2006). When plants were grown in absence of Fe at pH 7.5 the Fe stress was much more intense and the synthesis of phenolics in roots was fully enhanced (when compared with Fe-sufficient plants grown either at high or low pH): concentrations of all phenolics in roots were much higher (**Figure 5**; Supplementary Figure S4A), the concentration of phenolics in the nutrient solution increased markedly with time (**Figure 7**; Supplementary Figure S4A), and there were marked decreases in leaf Chl (**Figures 2A,B**), shoot biomass and shoot and root Fe contents (**Figure 2C**). The high pH/zero Fe effect is rapid, since only after 3 days roots already showed an increased expression of genes coding for root coumarin synthesis (*COMT, CCoAMT* and *F6′H1*) and Fe acquisition components (*IRT1* and *FRO2*) (when compared with Fe-sufficient plants grown either at high or low pH) (**Figure 2D**). In contrast, when plants were grown in absence of Fe at pH 5.5, there was no effect on biomass (**Figure 2C**) and the decreases in leaf Chl and shoot and root Fe contents (when compared with Fe-sufficient plants grown either at high or low pH) were as large as those found at high pH (**Figures 2A–C**), and only moderate effects were found with respect to phenolics, including: (i) increases of some phenolics in roots (fraxetin, isofraxidin, fraxinol, cleomiscosins A, C, and D) (**Figure 5**; Supplementary Figure S4A); (ii) time dependent increases in the concentration of all phenolics in the nutrient solution, although concentrations were always lower than those found at high pH (**Figure 7**; Supplementary Figure S4A), and (iii) a rapid (at 3 days) root increased expression of genes for Fe root uptake, although to a much lower extent than at high pH, without any change in the expression of genes involved in coumarin synthesis (**Figure 2D**).

Iron-supply and nutrient solution pH affect the relative coumarin concentrations in root extracts and growth media. Whereas the non-catechol coumarin scopoletin was initially the most abundant coumarin in root extracts and growth media, the catechol coumarin fraxetin was progressively more abundant with time in the growth media of plants grown with zero Fe (**Figure 6**). When other authors used HPLC-fluorescence for quantification, scopoletin was found to be the most abundant coumarin in the growth media of Fe-deficient *A. thaliana* (Schmid et al., 2014); fraxetin was not quantified in that study, possibly due to the very low fluorescence rate of this compound. The extremely low fluorescence of fraxetin in comparison with those of other coumarins (scopoletin, isofraxidin and esculetin) in the growth media of Fe-deficient *A. thaliana* plants is shown in Supplementary Figure S5. Interestingly, in the roots of Fe-deficient plants grown at pH 7.5 the coumarins that have a larger aglycone fraction (scopoletin and fraxetin; Supplementary Figure S4B), likely due to the action of a glucosidase, were also the prevalent ones in the growth media, supporting that the aglycone forms are likely to be the substrate for the plasma membrane transporter ABCG37. In this respect, the β-glucosidase BGLU42 is induced by Fe deficiency in roots (García et al., 2010; Yang et al., 2010; Lan et al., 2011; Rodríguez-Celma et al., 2013), and the roots of Fe-deficient *bglu42 A. thaliana* mutant plants apparently fail to secrete coumarins (Zamioudis et al., 2014). However, coumarin glucosides such as scopolin have been reported to occur in the exudates of Fe-deficient *A. thaliana* in other studies (Schmid et al., 2014; Schmidt et al., 2014).

The structural features of each coumarin-type compound may confer specific roles that contribute to the adaptation of *A. thaliana* to low Fe availability in alkaline conditions. The catechol moiety enable coumarins to mobilize efficiently Fe from an Fe(III)-oxide (**Figure 8A**). Fraxetin, a coumarin bearing a catechol moiety and a methoxy substituent, mobilized much more Fe than any of the non-catechol coumarins tested at the same concentration (100 μM; scopoletin, isofraxidin and fraxin) at physiologically relevant pH values (5.5 and 7.5). Specific structural features of the non-catechol coumarins tested, such as the *O*-glucosyl moiety (in fraxin) and one or two methoxy groups (in scopoletin/fraxin and ixofraxidin, respectively) do not appear to affect to the Fe mobilization ability of the coumarin, since these three coumarins mobilized similar amounts of Fe (**Figure 8A**). This confirms what has been reported previously (at pH 7.2) with the catechol coumarin esculetin (no methoxy substituent) and the non-catechol coumarins scopoletin (one methoxy and one hydroxy substituents) and esculin (one *O*-glucosyl and one hydroxy substituents) (Schmid et al., 2014). In addition, the present study revealed that the mobilization of Fe from Fe(III)-oxide promoted by fraxetin involves a significant reduction of Fe(III) to Fe(II) and appears to be controlled by the fraxetin concentration and the medium pH. Approximately 42% of the Fe mobilized by fraxetin was trapped by BPDS, regardless of the assay pH and the fraxetin concentration (**Figure 8**). The Fe(II) produced may be directly taken up by root cells, chelated by other natural ligands and/or re-oxidized to Fe(III). The amount of Fe mobilized by fraxetin was 1.6-fold higher at pH 7.5 -typical of calcareous soils- than at pH 5.5 (**Figure 8A**). Also, increases in fraxetin concentration (from 10 to 100 μM) led to a marked enhancement in Fe mobilization rates (**Figure 8B**). Most of the fraxetin produced by Fe-deficient plants (80–90%) was allocated to the nutrient solution regardless of the growth media pH, in contrast with the small amount of the non-catechol coumarin, scopoletin, allocated to the nutrient solution (12–23%) (**Figure 6B**). Taking also into account the concentrations estimated for scopoletin (21 μM), fraxetin (43 μM), isofraxidin (14 μM) and fraxinol (0.5 μM) in the soil solution surrounding the root (apex) of *A. thaliana* growing without Fe at pH 7.5 (calculated as in Römheld, 1991, for phytosiderophores), it seems likely that fraxetin could play a role as an Fe mobilizer in natural conditions. A catechol group is also present in the coumarinolignans 5′-hydroxycleomiscosins A and B (**Figure 1C**) that were found only in exudates (**Table 1**; **Figure 7**). Therefore, not only fraxetin but also 5-hydroxycleomiscosins A/B may have a role in mining Fe from soil Fe sources at high pH, providing soluble Fe for plant uptake. Unfortunately, no authenticated standards exist in the market for these compounds. On the other hand, coumarins, having or not catechol groups, play a well-established role in plant defense, serving as allelochemicals against a broad array of organisms (e.g., bacteria, fungi, nematodes, insects, etc), with their synthesis being activated in plants after infection (Weinmann, 1997; Bourgaud et al., 2006). Therefore, the array of coumarin-type compounds found in the growth media could play multiple roles, achieving different benefits for Fe-deficient plants.

Accumulating experimental evidences suggest that the Fe deficiency-elicited production of coumarin-type phenolics allows *A. thaliana* plants interacting with the rhizosphere microbiome, including beneficial and pathogen organisms. On one hand, Fe-deficient *A. thaliana* plants display reduced susceptibility to infection with the necrotrophic fungus *Botrytis cinerea* and the bacterial plant pathogen *Dickeya dadantii*, with an Fe supplementation restoring symptoms severity (Kieu et al., 2012). On the other hand, the activation of immunity toward broadly diverse pathogens and even insects and herbivores in *A. thaliana* elicited by the beneficial rhizobacteria *Pseudomonas fluorescens* WCS417 and mediated by the root-specific transcription factor MYB72 (Van der Ent et al., 2008; Segarra et al., 2009), also required for the induction of Fe deficiency responses (Palmer et al., 2013), involves not only the production of F6’H1-dependent coumarins but also their secretion (Zamioudis et al., 2014). In fact, two *Arabidopsis* mutants failing in the production and/or secretion of coumarins, *myb72* and *bglu42*, did not show, when grown in the presence of WCS417, enhanced resistance against two biotrophic pathogens (the Gram-negative bacterium *Pseudomonas syringae* pv. tomato DC3000 and the pseudo-fungus *Hyaloperonospora arabidopsidis*; Zamioudis et al., 2014). Also, BGLU42 overexpression led to a significantly enhanced resistance against *B. cinerea, H. arabidopsidis* and *P. syringae* pv. tomato DC3000 (Zamioudis et al., 2014). The enhanced disease resistance of *A. thaliana* against different pathogens can be associated with the structure of the coumarin-type compounds produced, since different substituents in the backbone of coumarins and lignans can influence biological activity (Weinmann, 1997; Apers et al., 2003; Borges et al., 2005; Zhang et al., 2014; Pilkington and Barker, 2015).

Certain structural features of coumarins and coumarinolignans produced by roots of Fe-deficient *A. thaliana* plants may confer specific roles in shaping the rhizosphere microbiome. In fact, the existence of differences in inhibitory potential against specific microorganisms may be expected in Fe deficiency-induced coumarins. First, all coumarins detected in Fe-deficient *A. thaliana* root extracts and exudates are highly oxygenated and with hydroxyl/methoxy substituents: scopoletin and esculetin are di-oxygenated and fraxetin, fraxetin isomer, isofraxidin and fraxinol are tri-oxygenated (**Figure 1A**). A high number of oxygen-containing substituents in the benzopyrone coumarin backbone (**Figure 1A**) appears to be determinant for broadening the antibacterial spectrum (Kayser and Kolodziej, 1999), whereas the presence of simple substituents (e.g., hydroxy, methoxy) instead of bulkier chains may aid bacterial cell wall penetration. Second, an oxygenation pattern consisting in two methoxy substituents and at least one additional hydroxyl substituent is present in the minor tri-oxygenated coumarins isofraxidin and fraxinol produced by Fe-deficient *A. thaliana* roots. This oxygenation pattern seems to confer to tri-oxygenated coumarins a strong and wide inhibitory activity against Gram-positive and Gram-negative bacteria (Kayser and Kolodziej, 1999; Smyth et al., 2009). Furthermore, the estimated concentrations of scopoletin, fraxetin, isofraxidin and fraxinol in the soil solution surrounding the root (apex) of *A. thaliana* growing without Fe at pH 7.5 (see above) are close or above the minimum inhibitory concentration of di- and tri-oxygenated coumarins against Gram-positive and Gram-negative bacteria (1.3-11.2 and 0.9-4.5 μM, respectively; Kayser and Kolodziej, 1999).

Regarding plant coumarinolignans, the current knowledge on their biological activities is mostly pharmacological, derived from the ethno-medical utilization of some plant species (Begum et al., 2010; Zhang et al., 2014; Pilkington and Barker, 2015). Known activities of cleomiscosins include liver protection, cytotoxicity against lymphocytic leukemia cells, immunomodulation, and others. In plants, the defense roles for conventional lignans have been studied, and certain structural features appear to affect the activities against specific organisms. First, coumarinolignans are more aromatic than conventional lignans, suggesting they may have a higher effectiveness. For instance, increased antifungal activities were observed when the phenyl ring in a monomeric phenylpropanoid derivative was replaced by naphthyl or phenanthryl rings, whereas no or very low antifungal activity is associated to the monomeric phenylpropanoid moieties in conventional lignans (Apers et al., 2003). Second, the occurrence of methoxy substituents in lignans appear confer stronger insecticide and fungicide activities, whereas the presence of polar substituents, especially hydroxy or glycoside groups, sometimes reduced them (Harmatha and Nawrot, 2002; Harmatha and Dinan, 2003; Kawamura et al., 2004). Since cleomiscosin structures differ in the methoxy and hydroxy substituents (**Figure 1C**), their possible insecticide and fungicide activities is likely to be different.

Results presented here highlight that Fe deficiency elicits the accumulation in roots and secretion into the growth media of an array of coumarin-type compounds, including coumarinolignans (cleomiscosins A, B, C, and D and the 5′-hydroxycleomiscosins A and/or B) and simple coumarins (scopoletin, fraxetin, isofraxidin and fraxinol) in *A. thaliana*. The phenolics response was much more intense when the plant accessibility to Fe was decreased and Fe status deteriorated, as it occurs when plants are grown in the absence of Fe at pH 7.5. The structural features of the array of coumarins and lignans produced and their concentrations in roots and growth media suggest that they may play dual, complementary roles as Fe(III) mobilizers and allelochemicals. Fraxetin, a catechol coumarin, was the most prominent coumarin found in the growth media of Fe-deficient *A. thaliana* plants grown at high pH and was especially effective in mobilization of Fe from an Fe(III)-oxide. In contrast, the rest of coumarins were non-catechols and were present in much lower concentrations, and therefore their role in mobilizing Fe is unlikely, although they can still be efficient as allelochemicals. Therefore, the production and secretion of phenolics by roots in response to Fe deficiency would promote an overall decrease in the competition for Fe in the immediate vicinity of roots, resulting in improved plant Fe nutrition. Results also suggest that Fe deficiency could be a good experimental model to understand the ecological dynamics of the biotic interactions in the plant rhizosphere.

## Author Contributions

AA-F, PF, and AA conceived and designed the experiments, PS-T conducted experiments, collected data, and drafted the manuscript, AL-V quantified phenolics, carried out Fe mobilization studies and made figures, AA, FG, J-FB, JA, and AA-F wrote, reviewed and edited the paper. All authors read and approved the final manuscript.

## Conflict of Interest Statement

The authors declare that the research was conducted in the absence of any commercial or financial relationships that could be construed as a potential conflict of interest.
