# Supplementary material for: Accumulation and Secretion of Coumarinolignans and other Coumarins in Arabidopsis thaliana Roots in Response to Iron Deficiency at High pH
Source: Front Plant Sci. 2016 Nov 23;7:1711. doi: 10.3389/fpls.2016.01711 (PMC5120119; doi:10.3389/fpls.2016.01711)
Supplement: Supplementary file 1 [file Data_Sheet_1.DOC]

Supplementary Material

# Accumulation and Secretion of Coumarinolignans and other Coumarins by *Arabidopsis thaliana* Roots in Response to Iron Deficiency at High pH

Patricia Sisó-Terraza^1^†, Adrián Luis-Villarroya^1^†, Pierre Fourcroy^2^, Jean-François Briat^2^, Anunciación Abadía^1^, Frédéric Gaymard^2^, Javier Abadía^1^, Ana Álvarez-Fernández^1^*

† These authors contributed equally to this work.

*** Correspondence:** Corresponding Author: ana.alvarez@eead.csic.es

The following Supplementary Data is available for this article:

**Fig. S1** Analysis of standards of a selected group of coumarin glucosides, coumarins and phenylpropanoids, and a lignan by HPLC-ESI-MS(TOF) using Elution program 2.

**Fig. S2** Analysis of standards of a selected group of flavonoids and a stilbene by HPLC-ESI-MS(TOF) using Elution program 2.

**Fig. S3** High-resolution MS^2^ spectra of compounds *14*-*18*.

**Fig. S4** Total concentrations of coumarins in root extracts and nutrien solutions, and relative concentrations of glycosides and aglycones in root extracts.

**Fig. S5** Emission spectra of scopletin, fraxetin, isofraxidin and other coumarins**.**

**Table S1** Coumarins produced by *Arabidopsis thaliana* roots in response to iron deficiency as reported in previous studies.

**Table S2** Elution programs used for HPLC-based separation.

**Table S3** List of eluents and standards used.

**Table S4** Operating conditions of HPLC-ESI-MS/MS(HCT Ultra ion trap).

**Table S5** Primers used for qRT-PCR.

**Table S6** Changes in young leaf photosynthetic pigment concentrations with Fe deficiency and growth media pH in Arabidopsis.

**Table S7** Changes in shoot and root Mn, Cu and Zn contents with Fe deficiency and growth media pH in Arabidopsis.

**Table S8** Mass losses, from the precursor ion, observed in the MS^2^ spectra of compounds *14*-*18*.

**1 Supplementary Figures**

**Figure S1** Analysis of a selected group of coumarin glucosides (a, b, and d), coumarins (c, f, i, j, k, o, and q) and phenylpropanoids (e, g, h, l, m, n, and p), and a lignan (r) by HPLC-ESI-MS(TOF). The standards were separated using Elution program 2. Chromatograms were extracted at the *m/z* (± 0.05) ratio corresponding to the [M+H]^+^ ions with the exception of those corresponding to glucosides (esculin, scopolin and fraxin) and the phenylpropanoid acids (caffeic and ferulic acid) for which the *m/z* (± 0.05) ratio corresponding to the [M-glucose+H]^+^ and [M-H_2_O+H]^+^ ions were used, respectively.

**Figure S2** Analysis of standards of a selected group of flavonoids (a, b, d, e, f, g, h, i) and a stilbene (c) by HPLC-ESI-MS(TOF). The flavonoids selected correspond to different classes: i) two flavonols (d, g) and their rhamnosides (b, e), ii) a flavanone (f), iii) a isoflavone (h) and its glucoside (a), iv) flavone (i). The standards were separated using Elution program 2. Chromatograms were extracted at the *m/z* (± 0.05) ratio corresponding to the [M+H]^+^ ions with the exception of those corresponding to the glucoside myricitrin and the rhamnosides (myricitrin and quercetrin) for which the *m/z* (± 0.05) ratio corresponding to the [M-glucose+H]^+^ and [M-rhamnose+H]^+^ions were used, respectively.

**Figure S3** High-resolution MS^2^ spectra of compounds *14*-*18* obtained on the HPLC-ESI-Q-TOF mass spectrometer in positive ion mode. The encircled numbers in each spectra correspond to the phenolic compounds listed in Table 1. In each spectra, the parent ion and the product ion used in later MS^3^ (Figure 4b) experiments are in bold blue colour and bold black colour, respectively.

**Figure S4** Effects of Fe deficiency, high pH and/or time on the total coumarin concentrations in *Arabidopsis thaliana* root extracts and nutrient solutions (a) and on the relative concentration of the aglycone and glycoside (hexoside) form for each coumarin in root extracts (b). Plants were pre-grown as indicated in Figure 2 and grown for 7 or 14 d with 0 (-Fe) or 20 µM Fe (+Fe) in nutrient solution buffered at pH 5.5 (with 5 mM MES-NaOH) or 7.5 (with 5 mM HEPES-NaOH). Data are means of n=3-5. The absolute values are shown in Figures 5 and 7.

**Figure S5** Emission spectra of a selected group of coumarins at wavelength of excitation of 360 nm. Solutions were prepared in water at pH 5.3. The spectra were recorded using a Perkin Elmer Luminescence Spectrometer Model LS-50B.

# Supplementary Tables

**Table S1** Coumarins accumulated and/or released into the growth media by roots of *Arabidopsis thaliana* (Col0, *pdr9-2*, *pdr9-3* and *comt*) in response to Fe deficiency as reported in previous studies ([1] Fourcroy et al. (2014), [2] Schmidt et al. (2014) and [3] Schmid et al. (2014)). N.D. means not detected. Table footnotes denote other names used for the cited coumarins. Col0, wild-type Arabidopsis, ecotype Columbia 0; *pdr9-2* and *pdr9-3*, two nonallelic T-DNA insertion mutants in the Arabidopsis *ABCG37* (*PDR9*) gene; *comt*, T-DNA insertion mutant in the Arabidopsis *COMT* gene encoding the caffeic acid/5-hydroxyferulic acid O-methyltransferase.

| Coumarin | Roots | Growth media | Reference |
| --- | --- | --- | --- |
| dihydroxyscopoletin hexoside^1^ | *pdr9-2* | Col0 | [1], [3] |
| fraxin^2^ | Col0 | N.D. | [2] |
| scopolin^3^ | Col0, *pdr9-2*, *pdr9-3* | Col0 | [1], [2], [3] |
| ferulic acid hexoside^4^ | Col0, *pdr9-2*, *pdr9-3*, *comt* | N.D. | [1] |
| hydroxymethoxyscopoletin hexoside^5^ | *pdr9-2* | N.D. | [1] |
| fraxetin^6^ | Col0, *pdr9-2*, *pdr9-3* | Col0 | [1], [2], [3] |
| isofraxetin^7^ |  | Col0 | [3] |
| scopoletin^8^ | Col0, *pdr9-2*, *pdr9-3* | Col0, *pdr9-3* | [1], [2], [3] |
| isofraxidin^9^ | Col0, *pdr9-2*, *pdr9-3* | Col0, *pdr9-3* | [1], [3] |
| fraxinol^10^ | Col0, *pdr9-2*, *pdr9-3* | Col0, *pdr9-3* | [1], [3] |
| esculetin^11^ | Col0 | Col0 | [2], [3] |

^1^Trihydroxymethoxycoumarin hexoside; ^2^fraxetin glucoside or 8-hydroxyscopoletin glucoside; ^3^scopoletin glucoside; ^4^precursor of scopletin; ^5^dihydroxydimethoxycoumarin hexoside; ^6^7,8-dihydroxy-6-methoxycoumarin; ^7^dihydroxymethoxycoumarin; ^8^7-hydroxy-6-methoxycoumarin; ^9^methoxyscopoletin isomer or 7-hydroxy-6,8-dimethoxycoumarin; ^10^methoxyscopoletin isomer or 6-hydroxy-5,7-dimethoxycoumarin; ^11^6,7-dihydroxycoumarin.

**Table S2** Elution programs used for HPLC based separation of phenolics. The mobile phase was built with two solvents: 0.1% of formic acid in water (A) and 0.1% formic acid in methanol (B). The elution program 1 was designed by Fourcroy et al. (2014).

| Elution program 1 | | Elution program 2 | |
| --- | --- | --- | --- |
| Time (min) | % solvent B | Time (min) | % solvent B |
| 0 | 15 | 0 | 15 |
| 3 | 15 | 45 | 50 |
| 15 | 55 | 48 | 55 |
| 25 | 55 | 50 | 55 |
| 30 | 15 | 53 | 15 |
| 45 | 15 | 60 | 15 |

**Table S3** List of chemicals used.

| Name | Quality grade | Manufacturer |
| --- | --- | --- |
| 2-propanol^1^ | LC-MS grade | Fluka |
| apigenin^1^ | ≥95% | Sigma |
| artemicapin C^2^ | ≥95% | Phytolab |
| caffeic acid^1^ | ≥99%, | Fluka |
| coniferyl alcohol^1^ | 98% | Aldrich |
| coniferyl aldehyde^1^ | 98% | Aldrich |
| esculetin^1^ | 98% | Aldrich |
| esculin^1^ | ≥98% | Fluka |
| ferulic acid^1^ | 99% | Aldrich |
| formic acid^1^ | 98% | Fluka |
| fraxetin^1^ | ≥98% | Aldrich |
| fraxin^2^ | ≥98% | Phytolab |
| fraxinol^2^ | ≥99% | Phytolab |
| genistein^1^ | ≥98% | Sigma |
| genistin^1^ | ≥97.5% | Sigma |
| isofraxidin^1^ | ≥95% | Sigma |
| lithium hydroxide^1^ | ≥98% | Sigma |
| matairesinol^1^ | ≥85% | Fluka |
| methanol^1^ | LC-MS grade | Fluka |
| myricetin^1^ | ≥96% | Sigma |
| myricitrin^1^ | ≥99% | Sigma |
| naringenin^1^ | ≥95% | Aldrich |
| nitric acid^1^ | TraceSELECT® | Fluka |
| quercetin^1^ | ≥98% | Sigma |
| quercitrin^3^ | 91.7% | HWI Analytik |
| resveratrol^1^ | ≥99% | Sigma |
| scopoletin^1^ | ≥99% | Sigma |
| scopolin^2^ | ≥95% | Phytolab |
| sinapic acid^1^ | ≥98% | Aldrich |
| sinapic aldehyde^1^ | 98% | Aldrich |
| sinapyl alcohol^1^ | 80% | Aldrich |
| umckalin^2^ | ≥95% | Phytolab |

^1^Chemicals purchased from Sigma-Aldrich (St. Louis, MO, USA). ^2^Chemicals purchased from PhytoLab GmbH & Co. KG (Vestenbergsgreuth, Germany). ^3^Chemical purchased from HWI Analytik GmbH (Ruelzheim, Germany).

**Table S4** Operating conditions of the HCT Ultra ion trap mass spectrometer used for identification of phenolics.

| Source | Electrospray |
| --- | --- |
| Nebulizer gas | N_2_ |
| Nebulizer gas pressure | 40 psi |
| Drying gas | N_2_ |
| Drying gas (N_2_) flow rate | 9.0 l min^-1^ |
| Drying gas temperature | 360 ºC |
| Polarity | negative or positive |
| Endplate voltage | -0.5 kV |
| Spray tip voltage | 4 kV (for negative) or -4 kV (for positive) |
| Skimmer voltage | 40 V (in negative) or -40 V (for positive) |
| Orifice voltage | 117.3 V (in negative) or -117.3 V (for positive) |
| Operation mode | Multiple Reaction Monitoring (MRM) |
| Target for MRM mode | 70,000 |
| Maximum accumulation time for MRM mode | 200 ms |
| Mass-to-charge ratio (*m/z*) range for MRM mode | 100-1000 u |
| Fragmentation amplitude for MS^2^ and MS^3^ | 0.5-1.0 V |
| Isolation width for MS^2^ and MS^3^ | 1.0 |
| Cutoff selection to precursor mass for MS^2^ and MS^3^ | 27.0% |

**Table S5 Primers used for qRT-PCR.**

| gene | ID | orientation | sequence |
| --- | --- | --- | --- |
| *PP2* | At1g13320 | FW | TAACGTGGCCAAAATGATGC |
|  |  | REV | GTTCTCCACAACCGCTTGGT |
| *CCoAMT1* | At4g34050 | FW | GCTCCTCCTGATGCACCAAT |
|  |  | REV | CCATCACCAACAGGGAGCAT |
| *COMT* | At5g54160 | FW | TGCTCCTTCTCATCCTGGTAT |
|  |  | REV | CACGCAATGTTCGTCACTCC |
| *F6’H1* | At3g13610 | FW | GCCTGATATCTGCAGGAATGAAA |
|  |  | REV | ACTCTAGAAGCCTCCTCACCA |
| *FRO2* | At1g01580 | FW | GCGACTTGTAGTGCGGCTATG |
|  |  | REV | CGTTGCACGAGCGATTCTTG |
| *IRT1* | At4g19690 | FW | CGGTTGGACTTCTAAATGC |
|  |  | REV | CGATAATCGACATTCCACCG |
| *ABCG37 (PDR9)* | At3g53480 | FW | GCGAAACTCAGAGCTTGTGA |
|  |  | REV | AGTGCGCCGAAGATCAAAGA |

**Table S6** Effects of Fe deficiency and high pH on the photosynthetic pigment concentrations (in µmol m^-2^) in *Arabidopsis thaliana* young leaves. Plants were pre-grown for 11 d in the presence of 20 µM Fe (III)-EDTA at pH 5.5, and then grown for 14 d in a medium with 0 (-Fe) or 20 µM (+Fe) Fe(III)-EDDHA in nutrient solutions buffered at pH 5.5 (with 5 mM MES-NaOH) and 7.5 (with 5mM HEPES-NaOH). Data are means ± SE (n=2-3). For each compound, significant differences among treatments (at p<0.05) are marked with different letters in the same row.

|  |  |  | +Fe | -Fe |  |  | +Fe | -Fe |
| --- | --- | --- | --- | --- | --- | --- | --- | --- |
|  |  |  | pH 5.5 | |  |  | pH 7.5 | |
|  | | | | | | | | |
| Neoxanthin |  |  | 8.5 ± 0.6 b | 3.4 ± 0.9 a |  |  | 8.3 ± 0.2 b | 4.3 ± 0.5 a |
| Violaxanthin |  |  | 10.4 ± 1.6 b | 4.4 ± 0.9 a |  |  | 10.0 ± 0.2 b | 4.7 ± 0.6 a |
| Antheraxanthin |  |  | 0.00 ± 0.00 a | 0.13 ± 0.08 a |  |  | 0.00 ± 0.00 a | 0.26 ± 0.17 a |
| Lutein |  |  | 39.6 ±1.7 b | 18.4 ± 4.4 a |  |  | 38.2 ± 1.4 b | 20.0 ± 1.5 a |
| β-carotene |  |  | 16.4 ± 1.0 b | 7.4 ± 2.4 a |  |  | 16.8 ± 0.6 b | 7.2± 0.9 a |
| Carotenoids / Chl total |  |  | 0.26 ± 0.01 a | 0.33 ± 0.04 a |  |  | 0.27 ± 0.01 a | 0.28 ± 0.01 a |

**Table S7** Effects of Fe deficiency and high pH on the Mn, Cu, and Zn contents (in µg metal plant^-1^) in *Arabidopsis thaliana* shoots and roots. Plants were pre-grown for 11 d in the presence of 20 µM Fe (III)-EDTA at pH 5.5, and then grown for 14 d in a medium with 0 (-Fe) or 20 µM (+Fe) Fe(III)-EDDHA in nutrient solutions buffered at pH 5.5 (with 5 mM MES-NaOH) and 7.5 (with 5mM HEPES-NaOH). Data are means ± SE (n=2-5). For each metal, significant differences among treatments (at p<0.05) are marked with different letters in the same row.

|  |  |  | +Fe | -Fe |  |  | +Fe | -Fe |
| --- | --- | --- | --- | --- | --- | --- | --- | --- |
|  |  |  | pH 5.5 | |  |  | pH 7.5 | |
| SHOOTS | | | | | | | | |
| Mn |  |  | 3.36 ± 0.42 ab | 4.62 ± 0.80 b |  |  | 2.03 ± 0.16 a | 2.3 ± 0.10 a |
| Cu |  |  | 0.19 ± 0.00 a | 1.22 ± 0.17 b |  |  | 0.18 ± 0.01 a | 0.45 ± 0.10 a |
| Zn |  |  | 2.90 ± 0.22 ab | 4.29 ± 0.56 b |  |  | 1.71 ± 0.06 a | 1.23 ± 0.09 a |
| ROOTS | | | | | | | | |
| Mn |  |  | 0.36 ± 0.11 a | 1.98 ± 0.92 ab |  |  | 5.09 ± 2.25 c | 4.02± 1.94 bc |
| Cu |  |  | 0.05 ± 0.01 a | 0.66 ± 0.42 a |  |  | 0.04 ± 0.00 a | 0.36 ± 0.20 a |
| Zn |  |  | 2.18 ± 0.70 a | 1.79 ± 0.25 a |  |  | 1.19 ± 0.28 a | 0.88 ± 0.02 a |

**Table S8** Mass losses of the MS^2^ ion trap product ions, from their corresponding precursor [M+H]^+^ ions, for the compounds *14*-*18* produced by *Arabidopsis* *thaliana* roots in response to Fe deficiency. The numbers in italics (#) refer to the labels used for each compound in Figure 3 and Table 1. Common product ions and mass losses among the MS^2^ spectra of *15*-*18* are indicated in bold.

| # | *14* | | *15* | | *16* | | *17* | | *18* | |
| --- | --- | --- | --- | --- | --- | --- | --- | --- | --- | --- |
| Annotation | 5’-Hydroxycleomiscosins A and/or B | | Cleomiscosin D | | Cleomiscosin C | | Cleomiscosin B | | Cleomiscosin A | |
| Parent ion *m/z* | 403 | | 417 | | 417 | | 387 | | 387 | |
|  | Product ions *m/z* | Mass loss (Da) | Product ions *m/z* | Mass loss (Da) | Product ions *m/z* | Mass loss (Da) | Product ions *m/z* | Mass loss (Da) | Product ions *m/z* | Mass loss (Da) |
|  | 385 | 18 | 387 | **30** | 387 | **30** | 357 | **30** | 357 | **30** |
|  | 373 | 30 | 367 | **50** | 367 | **50** | 337 | **50** | 337 | **50** |
|  | 279 | 124 | 263 | 154 | 263 | 154 | 263 | 124 | 263 | 124 |
|  | 251 | 152 | 233 | 184 | 233 | 184 | 233 | 154 | 233 | 154 |
|  | 221 | 182 | 221 | 196 | 221 | 196 | **209** | 178 | **209** | 178 |
|  | 209 | 194 | **209** | 208 | **209** | 208 | 161 | 226 | 223 | 164 |
|  | 151 | 252 | 193 | 224 | 192 | 225 | 131 | **256** | 162 | 225 |
|  | 137 | 266 | 161 | **256** | 161 | **256** |  |  | 131 | **256** |
|  |  |  | 133 | 284 | 131 | 286 |  |  |  |  |

**
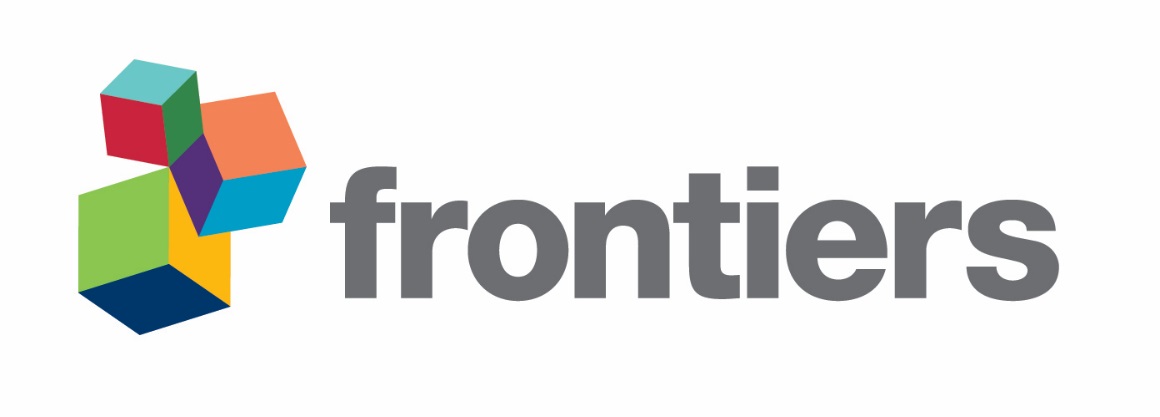
**
